# Treatment in acute HIV infection only temporarily preserves monocyte function: a comparative cohort study in adult males

**DOI:** 10.1016/j.ebiom.2025.105997

**Published:** 2025-11-07

**Authors:** Killian E. Vlaming, Pien M. van Paassen, John L. van Hamme, Stella Schonherr, Tanja M. Kaptein, Karel van Dort, Irma Maurer, Reinout van Crevel, Casper Rokx, Liffert Vogt, Jan M. Prins, Neeltje A. Kootstra, Teunis B. Geijtenbeek, Godelieve J. de Bree, NOVA study team, Godelieve De Bree, Jan M. Prins, Annelies Verbon, Liffert Vogt, Peter Reiss, Casper Rokx

**Affiliations:** a Amsterdam UMC Location University of Amsterdam, Department of Experimental Immunology, Meibergdreef 9, Amsterdam, the Netherlands; b Amsterdam Institute for Infectious Diseases and Immunology, Amsterdam, the Netherlands; c Department of Internal Medicine and Radboud Center for Infectious Diseases, Radboud University Medical Center, the Netherlands; d Department of Internal Medicine and Department of Medical Microbiology and Infectious Diseases, Erasmus University Medical Center, Rotterdam, the Netherlands; e Apheresis Unit, Dianet, Location Amsterdam UMC, Amsterdam, the Netherlands; f Department of Internal Medicine, Division of Infectious Diseases, Amsterdam UMC, Amsterdam, the Netherlands

**Keywords:** HIV-1, Monocytes, Toll like receptor 8, RIG-I like receptors, Innate immunity

## Abstract

**Background:**

Persistent monocyte activation and altered cytokine responses are reported in PWH despite ART. How prior HIV-1 infection status and timing of ART initiation relate to monocyte pattern-recognition receptor crosstalk between TLR8 and RLRs remains uncertain.

**Methods:**

We conducted a comparative cohort study in adult males enrolled from two Dutch HIV-cohorts. Participants included HIV-negative participants, PWH who initiated ART during chronic HIV infection, and PWH who initiated ART during acute HIV infection, with sampling at 24 and 156 weeks after ART initiation for the acute group. PBMCs were stimulated with an RLR agonist, a TLR8 agonist, or both. Monocyte surface markers were assessed by flow cytometry and pro-inflammatory cytokines were analysed with qPCR and ELISA.

**Findings:**

Across groups, RLR stimulation induced IL-12p70 and IL-27, TLR8 stimulation induced IL-6 and IL-12p70 and combined TLR8 + RLR co-stimulation synergistically increased IL-12p70 and IL-27 while restricting IL-6. Compared with controls, CHI showed reduced IL-12p70 and IL-27 and higher IL-6. In AHI at 24 weeks, cytokine patterns and co-stimulation effects resembled HIV-negative participants; by 156 weeks, responses were attenuated and approximated CHI.

**Interpretation:**

In this male cohort, TLR8–RLR crosstalk was preserved early after ART initiation during acute infection but diminished over time, approaching profiles observed in chronically treated infection. These observations emphasise a potential early window after ART initiation for interventions aiming to preserve monocyte function and motivate studies to characterise underlying mechanisms.

**Funding:**

Funding for this study was obtained through a ZonMW/Aidsfonds grant NL4Cure: Bridging shock and kill strategies (446002508).

**Research in context**

**Evidence before this study**

Prior studies showed that persistent immune activation and monocyte dysfunction continue in people with HIV despite viral suppression, and that early antiretroviral therapy (ART) can only partially restore monocyte function. Our previous work in HIV-uninfected individuals, in absence of ART, demonstrated that crosstalk between Toll-like receptor 8 (TLR8) and RIG-I-like receptors (RLR) results in an enhanced antiviral cytokine profile, evidenced by increased IL-12 and IL-27 production in monocytes while limiting IL-6. Given the persistent monocyte dysfunction observed in HIV-positive individuals despite ART, it remained unclear how this dysfunction might influence the TLR8/RLR crosstalk.

**Added value of this study**

Although starting ART during acute HIV infection temporarily maintains the TLR8/RLR-driven antiviral cytokine profile, this advantage is lost progressively, resulting in cytokine profiles that resemble the diminished crosstalk that we observe in chronic HIV infection. Our findings highlight the transient nature of monocyte function preservation in acutely treated HIV infection.

**Implications of all the available evidence**

Given their role in steering the antiviral immune response, a preserved monocyte function is critical for durable antiviral immunity. Such antiviral immunity is potentially critical in the design of curative HIV treatment strategies that aim to enhance immunity and viral control after ART interruption. Specifically interventions aimed at enhancing monocyte functionality can help reduce inflammation and improve long-term immunological health in HIV-1. The implication of the present study is that monocyte functionality declines progressively, suggesting a critical early window of opportunity shortly after ART initiation for interventions aimed at preserving monocyte function. Nonetheless, later interventions designed to restore this lost functionality remain an important area for further investigation.

## Introduction

Immune activation is a hallmark of HIV infection and clinically significant since it is associated with poorer CD4+ T cell recovery and non-AIDS related morbidity development in people with HIV (PWH) despite effective anti-retroviral treatment.^1^, ^2^, ^3^ Part of HIV associated immune activation is reflected by persistent monocyte activation, and elevated levels of soluble markers associated with monocyte activation are observed in PWH on ART.^4^ Apart from the broader immune activation, monocyte activation in the acute phase of HIV infection (AHI) involves changes in expression of interferon-regulated genes, this process is only partially restored by initiation of ART.^5^ These data suggest persistent changes in monocyte function despite viral suppression.^4^^,^^6^

Monocytes are a pivotal component of the innate antiviral defence and shaping adaptive immunity. Specifically, the role of monocytes in HIV infection was demonstrated in nonhuman primate models using simian immunodeficiency virus (SIV). Here blocking type I interferon during the acute phase can partially alleviate the pro-inflammatory state and enhanced T cell responses.^7^^,^^8^ This emphasises the importance of understanding the characteristics of monocyte function and how these impacts downstream immunity in people with HIV. Key players in activation of monocytes are pattern recognition receptors (PRR). In earlier studies PPR agonists, which mimic PPR receptor engagement by viruses, have emerged as potential tools to induce immune activation.^9^^,^^10^ TLRs and RLRs are PRRs that can detect viral pathogen-associated molecular patterns (PAMPs), such as HIV RNA.^11^, ^12^, ^13^, ^14^ Activation of TLRs and RLRs triggers monocytes and results in the production of pro-inflammatory and immune modulating cytokines, like IL-6 and IL-12, type I interferons and interferon-stimulated genes (ISGs).^10^ We recently showed that crosstalk between two PRRs; TLR8 and RLRs, enhanced immunity.^15^ TLR8 is predominantly expressed in monocytes, macrophages, and dendritic cells and plays a crucial role in sensing single-stranded RNA, including HIV RNA.^16^^,^^17^ RLRs are cytosolic sensors of viral RNA that also trigger antiviral responses.^18^ We showed that the combined activation of TLR8 and RLRs in monocytes from HIV uninfected individuals led to an enhanced monocyte activation. This enhanced response was characterised by elevated production of IL-12p70 and IL-27 and a reduction in IL-6, indicative of a shift toward a more effective antiviral immune profile.^15^ Here we investigate whether the monocyte responses elicited by TLR8 and RLR crosstalk is affected in PWH. Furthermore, we evaluate the impact of the timing of ART initiation on TLR8 and RLR crosstalk in PWH by investigating these responses at different stages of infection; CHI, and AHI at 24 and 156 weeks after treatment initiation (AHI-24 and -156) as compared to HIV uninfected individuals.

Our findings show that the synergistic crosstalk between TLR8 and RLRs in monocytes is reduced in PWH who initiated ART during chronic infection as compared to HIV uninfected individuals. Notably, in PWH who started ART after AHI no monocyte dysfunction was observed at 24 weeks after treatment initiation and crosstalk between TLR8 and RLRs was comparable to those in HIV uninfected individuals. However, by 156 weeks post-treatment initiation, monocyte responses to combined TLR8 and RLR stimulation were decreased to levels similar to those observed in chronic HIV infection (CHI) and distinct from those from healthy controls. These results suggest that ART initiation in acute infection only temporarily preserves monocyte function, and over time, monocyte dysregulation emerges despite continued viral suppression.

## Methods

### Study population

For this study 78, all male, participants were enrolled from two HIV cohorts, the Amsterdam Cohort Studies (ACS) and Netherlands Cohort Study on Acute HIV Infection (NOVA).

56 participants were selected from the ACS,^19^^,^^20^ criteria for selection was based on the known size of their viral reservoir to allow us to correlate observed immune responses to the viral load. Age-matched HIV-negative participants from the same cohort were selected randomly to ensure comparison. The ACS is an MSM cohort in Amsterdam where participants are routinely sampled longitudinally, 28 had seroconverted for HIV prior to inclusion in the current study, with antiretroviral treatment being started during the chronic phase of disease (CD4 230 (IQR 130-380) × 10E^6^/L), in accordance with treatment protocols at the time. The other 28 participants had not seroconverted prior to their inclusion and served as HIV negative participants and comparators to the HIV-positive individuals. All ACS participants were virally suppressed at inclusion in the study and samples were selected under request code 2023-3. The ACS cohort study is described in detail in de Wolf et al.^19^

22 participants were selected from the NOVA cohort from two distinct timepoints, at 24 weeks post start treatment (PST) and at 156 weeks PST. The NOVA is a multicentre, observational, prospective cohort that was initiated in 2015 and includes participants diagnosed with an acute/early HIV infection (AHI).^21^ People are included upon HIV-diagnosis during AHI as defined by Fiebig stage I-IV.^21^^,^^22^ In case of positive Western blot, participants can be included if there is a documented negative HIV screening test <6 months before inclusion. Post diagnosis, participants are referred to an HIV treatment centre to start antiretroviral treatment as soon as possible. The participants selected for the current analysis were in care at the Amsterdam University Medical Center, Erasmus Medical Center or Radboud University Medical Center. All NOVA participants were virally suppressed, at the selected 24 weeks and 3 year timepoints, with the exception of two participants, who showed viral blips: 74 and 98 copies/mL (in graphs marked with ▲), and in one donor and 200 → 100 copies/mL (in graphs marked with ▼) respectively and samples were selected under request code 11072022. The NOVA cohort study is described in detail in Dijkstra et al.^21^ The race and ethnicity of all participants was not known at the time of inclusion.

### Ethics

The ACS has been conducted in accordance with the ethical principles set out in the declaration of Helsinki. Authors had no access to information that could identify individual participants during or after data collection. Written informed consent was obtained from all participants. The ACS was ethically approved by the institutional review board (METC) of the Academic Medical Center (renewed approval METC 07-182). The NOVA cohort study received ethical approval under protocol METC 2014_371.

### Cell culture

PBMCs were stored in liquid nitrogen prior to thawing. Cells were incubated post-thaw with DNase (2.5 U/10^7^ cells) for 2 h at 37 C after which survival was determined using trypan-blue staining. Subsequently cells were cultured in RPMI medium enriched with 10% FCS (Biological Industries), 10 IU/mL penicillin (Thermo Fisher), 10 mg/mL streptomycin (Thermo Fisher), 2 mM l-glutamine (Lonza) and 10 IU/mL IL-2 (Invivogen). Cells were plated in round bottom plates in 200.000 PBMCs per condition in technical triplicate and left overnight at 37 C prior to stimulation.

Subsequently, in case of plentiful PBMCs, monocytes were isolated from remaining PBMCs with magnetic selection using CD14+ microbeads (Miltenyi) to isolate a pure monocyte population. Subsequently monocytes were subsequently cultured in RPMI medium enriched with 10% FCS (Biological Industries), 10 IU/mL penicillin (Thermo Fisher), 10 mg/mL streptomycin (Thermo Fisher), 2 mM l-glutamine (Lonza) overnight before being lysed for mRNA extraction.

Cells were thawed in batches due to practical limitations. CG and CHI samples were initially thawed and mixed to avoid batch bias. Following analysis of CG and CHI samples, AHI-samples were thawed, mixing 24 and 156 timepoints.

Paired AHI-samples shown in Fig. 4 were analysed freshly after isolation from blood through leukapheresis. Soluble markers were analysed in either plasma or serum, directly after leukapheresis. Monocyte subsets were determined prior to cryopreservation of material.

### Stimuli used

RLR agonist PolyIC-lyovec (Invivogen) and dissolved in LAL-water as per the manufacturer's instructions and used at a concentration of 2 μg/mL. TLR8 agonist Selgantolimod (GS9688) was dissolved in DMSO at 10 mM concentration and used at a concentration of 1 μM. Salmonella Lipopolysaccharide (LPS) was used as positive control at a concentration of 10 ng/mL.

All stimulations with PBMCs were performed in technical triplicates, cells were incubated with stimuli for 24 h after which supernatant was removed and stored for analysis. Triplicates were subsequently pooled for flow cytometry.

### ELISA

Supernatant of PBMCs were harvested at select timepoints after stimulation. Subsequently secretion of IL-6, IL-12p70 and IL-27 proteins were measured by ELISA as described by the manufacturer (IL-6, IL-12p70 eBiosciences, IL-27 UcyTech). OD450 nm values were measured using BioTek synergy HT. Concentrations of I-FABP, IL-6, sCD14, and sCD163 were determined in plasma samples stored at −80 °C using enzyme-linked immunosorbent assay (ELISA) (I-FABP, IL-6, CD14, and CD163 DuoSet ELISAs; R&D Systems).

### Quantitative real-time PCR

mRNA was acquired following lysis of cellular material at 8 h, it was transcribed to cDNA using a reverse transcriptase kit (Promega). Quantitative real-time PCR was performed on an ABI 7500 Fast real-time PCR detection system from Applied Biosystems using SYBR green (Thermo fisher). Expressions of genes of interests were normalised to a household gene (GAPDH). Although RNA integrity numbers (RIN) were not formally measured, robust amplification of housekeeping gene (GAPDH) in all samples indicated adequate RNA integrity.

The formula used was Nt = 2^Ct(GAPDH)−Ct(target)^. Expression was subsequently normalised to expression of the TLR-stimulus at T = 8 h.

### Flow cytometry

Following stimulation PBMCs were, stained at 4 C in the dark for 30 min with PE Cy5.5 conjugated anti-CD14 (1:100, Biolegend, RRID AB_893250), APC-Cy7 conjugated anti-CD16 (1:100, Biolegend, RRID AB_314218), FITC conjugated anti-CD86 (1:25, Biolegend, RRID AB_2721574), PE conjugated anti-CD80 (1:25, Biolegend, RRID AB_2890803), PE-Cy7 conjugated anti-CD64 (1:100 Biolegend, AB_2051583), APC conjugated anti-CD163 (1:50, Biolegend, AB_2564015), Alexa-fluor 700 conjugated anti-CD11b (1:100, Biolegend, AB_2750074) and PE-Dazzle conjugated anti-HLA-DR (1:100, Biolegend. AB_2563646).

After staining, cells were washed twice using PBS and fixed or 15 min using 4% PFA. Cells from CG, AHI and CHI groups were taken up in FACS-PBA and analysed on a BD Canto II, 2 laser system (488, 637 nm) (CG, CHI), and following technical difficulties a BD Symphony A1, 4 laser system (405, 488, 561, 637 nm) (AHI-24/AHI-156). No dedicated live-dead marker was included, cells were gated using a lymphocyte based on FCS/SCC.

Additional Monocyte analyses (Fig. S3) for paired AHI samples were analysed on a BD Canto II, 3 laser system (405, 488, 637 nm) in a BSL-3 facility. Fresh PBMCs were obtained following leukapheresis and separated by ficoll density gradient, then stained at 4 C in the dark for 30 min with Pe-Cy7 conjugated anti-CD14 (1:500, Biolegend, AB_2566714), and eFluor 450 conjugated anti-CD16 (1:20, Thermofisher eBioscience, AB_1272052). All antibody concentration were determined prior use via titration.

### Statistics

Two-way ANOVA tests were performed to compare data between donors when technical triplicates were obtained. One-way ANOVA tests were performed to analyse data between donors when only a single measurement was available. Comparisons between donors groups was performed using unpaired tests, comparisons between test conditions within the same donor was performed using paired tests. Statistical significance was set at ∗p < 0.05, ∗∗p < 0.01, ∗∗∗p < 0.001, ∗∗∗∗p < 0.0001. Statistical analysis of obtained data was performed using Graphpad Prism 10 (Graphpad Software Inc). All ANOVA analyses were followed by Tukey's post-hoc tests to adjust for multiple comparisons and control the family-wise error rate. In Fig. 2, as many tests were applied simultaneously, Benjamini-Hochberg's FDR correction was applied.

### Role of funders

Funding for this study was obtained through a ZonMW/Aidsfonds grant NL4Cure: Bridging shock and kill strategies (446002508). The funders had no role in study design, data collection, data analyses, interpretation, or writing of report. The authors were not involved with the funders.

## Results

### Study population characteristics

The baseline characteristics of the three participant groups—HIV-1 uninfected controls (CG), individuals treated during chronic HIV infection (CHI), and those treated during acute HIV infection (AHI), are summarised in Table 1. Of note, for ten participants in the AHI group, paired longitudinal samples from both 24 weeks (AHI-24) and 156 weeks (AHI-156) post-treatment initiation were available. The baseline characteristics of the study participants are provided in Table 1. All samples of PWH were virally supressed at the time points of investigation, with the exception of two donor in AHI-24. Comorbidities not clinically present at the time of the investigation, such as subclinical viral infections, are unknown.

**Table 1:** Baseline characteristics of participants included in this study.

Demographics	CG	AHI	CHI
Number of participants	28	22	28
Sex	100% male	100% male	100% male
Age at sampling in years (median; IQR)	45 (30–45)	38 (28–48)	42.5 (37–65)
Months since seroconversion (median; IQR)	n/a	n/a	30.5 (25–88)
**Country of birth**			
The Netherlands	28	18	25
Other		4	3
**Fiebig stage at diagnosis**^22^	n/a		n/a
Fiebig II		1	
Fiebig III/IV		12	
Fiebig V		5	
Fiebig VI		4	
Plasma viral load at sampling, × 10^6^ copies/ml (median; IQR)	n/a	Undetectable	Undetectable
CD4 T-cell count at start ART, × 10^6^ cells/L (median; IQR)	n/a	445 (308–593)	230 (130–380)
CD4 T-cell count at inclusion, × 10^6^ cells/L (median; IQR)	n/a	24 wk: 625 (535–755) 156 wk: 720 (630–860)	540 (385–658)
**Subtype HIV**	n/a		
B		15	28
CRF_02AG		1	0
AE		3	0
F		2	0
Unknown		1	0
**ART regimen**	n/a		
NRTI combination/PI		2	24
NRTI combination/NNRTI/PI			4
NRTI combination		20	
**Time on ART at inclusion in months** (median; IQR)	n/a	n/a	24 (22–25)

Numbers are n unless otherwise indicated. ART regimen: NRTI, Nucleoside Reverse Transcriptase Inhibitor; NNRTI, Non-Nucleoside Reverse Transcriptase Inhibitor; PI, Protease Inhibitor.

### Monocytes have a different activation profile following early or late initiation of ART

**Fig. 1:**
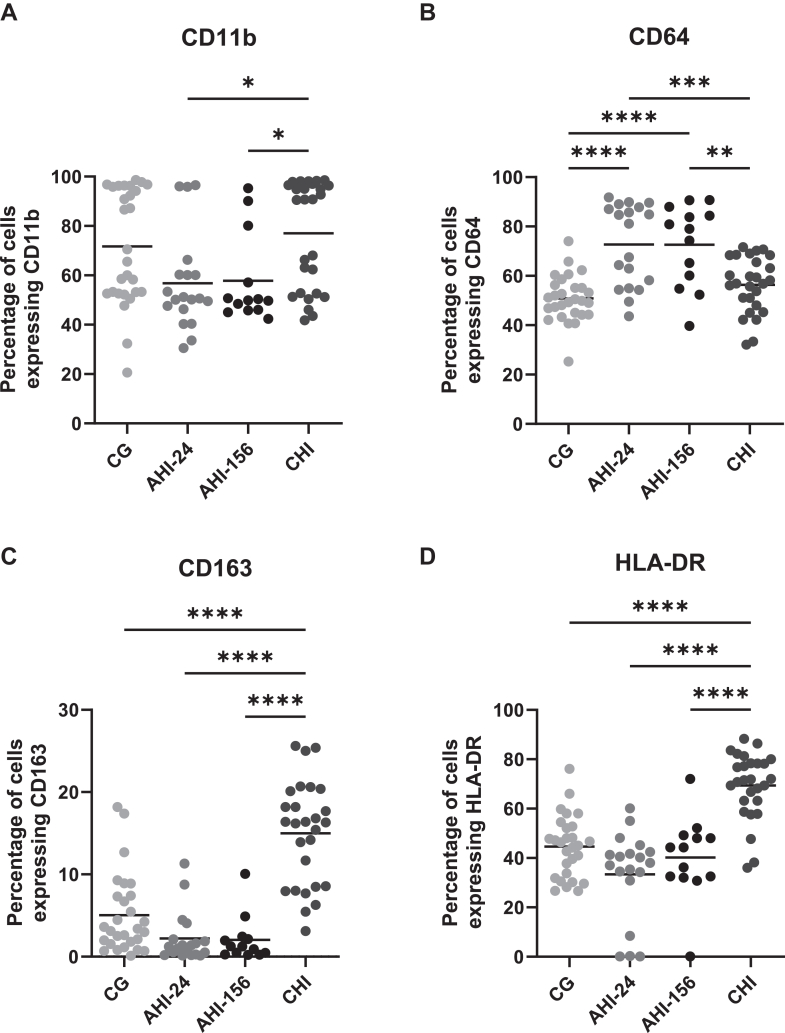
**Monocytes have a different activation profile following early or late initiation of ART**. (A–D) Surface expression of CD11b, CD64, CD163, and HLA-DR was assessed on monocytes from CG, CHI, AHI-24, and AHI-156 groups. Cells were thawed, stained for the indicated markers and analysed. Positive and negative samples were determined by single staining. CG n = 28, AHI-24 n = 19, AHI-156 n = 13, CHI n = 28, a single biological replicate was used per datapoint, error bars show SD. (A) CD11b expression was increased in all groups compared to CG, significantly so in AHI-24. (B) CD64 showed significant elevation across all HIV-infected groups, suggesting enhanced antibody-dependent phagocytosis. (C and D) CD163 and HLA-DR, associated with tissue repair and antigen presentation, were significantly elevated in the CHI group compared to other groups. Statistical analysis was performed using one-way ANOVA with Tukey's post-hoc correction. Significance is indicated as ∗p < 0.05, ∗∗p < 0.01, ∗∗∗p < 0.001, ∗∗∗∗p < 0.0001.

To investigate the impact of ART initiation in acute versus chronic infection on monocyte activation, we analysed the expression levels of surface markers CD11b, HLA-DR, CD64 and CD163, on monocytes from the three study groups. These markers were selected based on their roles in monocyte activation and function (Table S1). For this analysis, we focused on CD14 expressing monocytes (encompassing both classical CD14 + CD16-, and intermediate CD14+CD16+ monocytes), represent the major monocyte population and primary responders to inflammatory stimuli. Monocytes in both AHI-24, AHI-156 and CHI groups exhibited altered expression levels of activation markers compared to HIV negative individuals. In the CHI group, monocytes had significantly higher expression levels of CD163 and HLA-DR. CD11b expression in CHI was significantly elevated compared to both AHI groups, though not significantly different from CG. CD64 expression in CHI was not significantly different from CG (Fig. 1A–D).

In the AHI groups, monocytes showed significantly elevated CD64 expression compared to CG. Additionally, CD64 expression in both AHI groups was significantly lower than in CHI. The expression levels of HLA-DR and CD163 in both AHI-24 and AHI-156 were comparable to those observed in HIV negative individuals and significantly lower than in the CHI group, suggesting that early ART initiation mitigates the upregulation of these activation markers (Fig. 1C and D). Statistical analysis was performed using one-way ANOVA with Tukey's post-hoc correction. No dedicated live/dead viability dye was included in this panel, which limits some conclusions.

### Upregulation of pro-inflammatory cytokines in monocytes from CHI

To further explore functional properties of monocytes, we assessed the transcriptional profile of pro-inflammatory and interferon-stimulated genes (ISGs) in the three study groups. To achieve this, we investigated transcription of genes linked to inflammation, antiviral defences, or known roles in HIV-1 infection and immunity (Table S2).^23^, ^24^, ^25^, ^26^, ^27^, ^28^, ^29^, ^30^

**Fig. 2:**
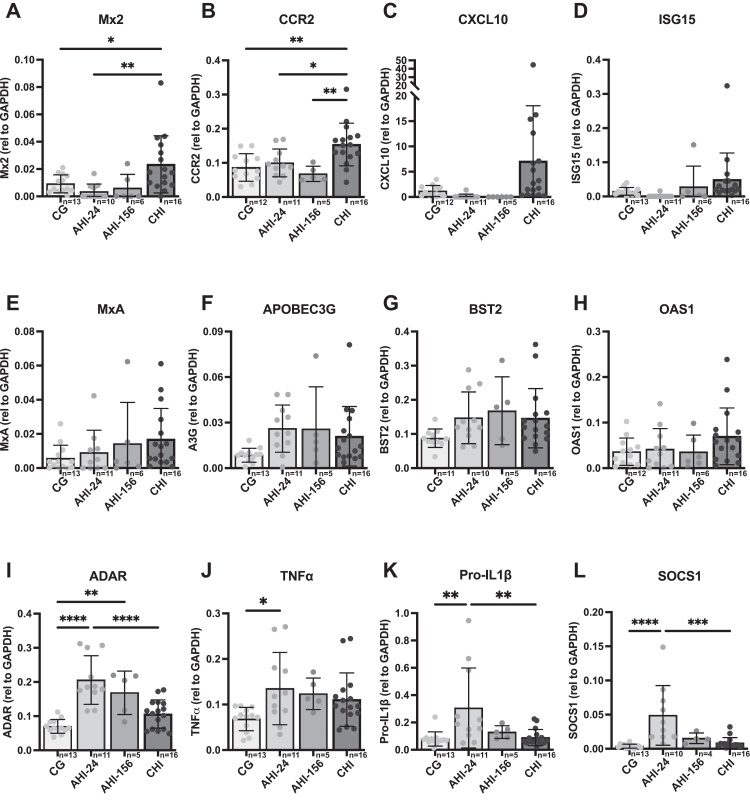
**Upregulation of pro-inflammatory cytokines in monocytes from CHI**. Monocyte mRNA levels of selected pro-inflammatory cytokines and ISGs were assessed by qPCR following isolation from PBMCs of CG, CHI, AHI-24, and AHI-156 individuals. (A–H) ISGs (Mx2, ISG15, CCR2, OAS1, and CXCL10) showed upregulation in the CHI group compared to both AHI and CG, indicating persistent immune activation. N shown on individual graphs (CG n = 13–12, AHI-24 n = 11–10, AHI-156 n = 6–5, CHI n = 16), a single biological replicate was used per datapoint, error bars show SD. (I–L) Pro-inflammatory markers (ADAR, TNFα, pro-IL-1β, and SOCS1) were elevated in the AHI-24 group, with decreased expression in AHI-156, suggesting a level of immune normalisation over time. Statistical analysis was performed using one-way ANOVA with Tukey's post-hoc correction. Significance is indicated as ∗p < 0.05, ∗∗p < 0.01, ∗∗∗p < 0.001, ∗∗∗∗p < 0.0001.

In the CHI group, monocytes exhibited significantly elevated mRNA levels of several ISGs, including Mx2, CCR2, compared to both CG and the AHI group at 24- and 156-weeks post ART initiation (Fig. 2A–H). In CHI as well as AHI-24 and AHI-156, we observed a trend toward increased mRNA levels of ISGs such as MxA, ISG15, CXCL10, APOBEC3G, and BST2 compared to CG, although these differences did not reach statistical significance (Fig. 2E–G). The persistent upregulation of several antiviral and inflammatory mRNA transcripts is suggestive of sustained monocyte activation during ART treated chronic HIV infection. In the AHI 24 weeks post ART, there was a transient upregulation in mRNA levels of the pro-inflammatory cytokine pro-IL-1β, as well as the regulatory molecule SOCS1. This changed at 156 weeks post ART the expression profile of pro-ILβ and ADAR were comparable to CHI. TNFα mRNA levels were higher in AHI-24, AHI-156 and CHI compared to HIV-negative controls, but not significantly so (Fig. 2I–L). Notably, at 156 weeks post-treatment initiation (AHI-156), mRNA levels of both pro-IL-1β and SOCS1 decreased to levels comparable to HIV uninfected individuals. Statistical analysis was performed using one-way ANOVA with Tukey's post-hoc correction. Significance is indicated as ∗p < 0.05, ∗∗p < 0.01, ∗∗∗p < 0.001, ∗∗∗∗p < 0.0001.

These findings indicate that the CHI group exhibited sustained upregulation of pro-inflammatory and antiviral genes, indicating chronic immune activation that differs from the pattern observed in monocytes from AHI at 156 weeks. In contrast, the AHI group exhibited transient upregulation of inflammatory markers at 24 weeks. By 156 weeks post-treatment initiation, although mRNA levels of pro-IL-1β and SOCS1 had decreased substantially, expression of pro-IL-1β, TNFα, ADAR, and SOCS1 remained elevated relative to both the CG and CHI groups, suggesting partial but incomplete normalisation.

### Prolonged HIV-1 infection is associated with reduced IL-12 and IL-27 cytokine responses to TLR8 and RLR agonists

We investigated whether the timing of ART initiation affects the ability of monocytes to produce antiviral cytokines, specifically IL-12p70 and IL-27, in response to stimulation with poly (I:C)-lyovec, a RLR agonist, a TLR8 agonist (GS-9688), or TLR8 in combination with RLR. Lastly, a control condition with LPS was included (Fig. S4).

**Fig. 3:**
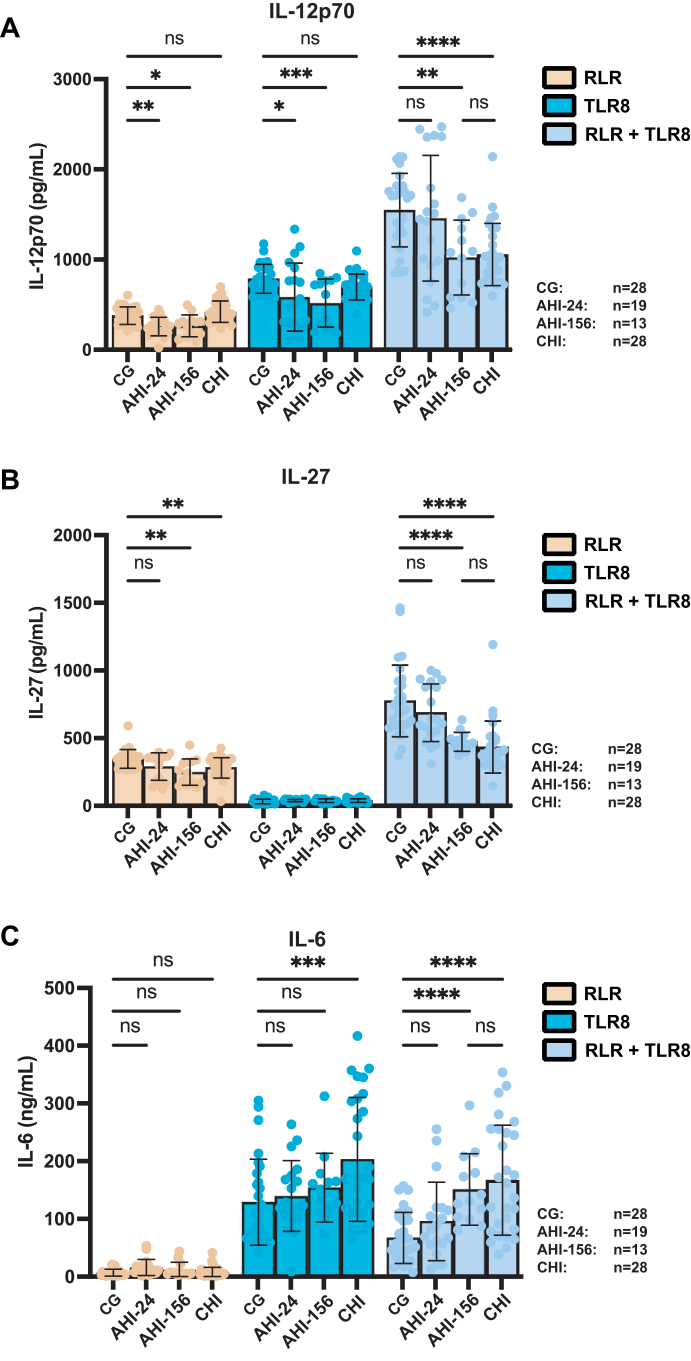
**Treatment initiation in chronic HIV-1 infection is associated with reduced IL-12 and IL-27 cytokine responses to TLR8 and RLR agonists**. PBMCs from CG, AHI-24, AHI-156, and CHI individuals were stimulated with TLR8 and RLR agonists, and (A) IL-12-70, (B) IL-27 and (C) IL-6 secretion in the supernatant after 24 h were assessed by ELISA. CG n = 28, AHI-24 n = 19, AHI-156 n = 13, CHI n = 28. Three biological replicates were used per datapoint. Error bars show SD. Statistical analysis was performed using unpaired two-way ANOVA with Tukey's post-hoc correction. Significance is indicated as ∗p < 0.05, ∗∗p < 0.01, ∗∗∗p < 0.001, ∗∗∗∗p < 0.0001.

Stimulation of PBMCs with the RLR agonist induced IL-12p70 across all groups (Fig. 3A). However, the production of IL-12p70 in AHI-24 and AHI-156 groups was significantly lower compared to CG. In contrast, the CHI group had IL-12p70 levels comparable to CG. Stimulation with the TLR8 agonist led to an induction of IL-12p70 in all groups. The level of IL-12p70 in AHI-24 and AHI-156 groups was lower compared to CG. The CHI group showed a slight, non-significant reduction compared to CG (Fig. 3A). Following LPS stimulation, IL-12p70 was induced in all groups, however, while induction between AHI-156 and CHI-groups was similar, levels if IL-12p70 induced in both CG and AHI-24 groups induced slightly higher, but significant, levels of IL-12p70 (Fig. S4B).

Combined stimulation with both TLR8 and RLR agonists resulted in a synergistic increase in IL-12p70 production in all groups. IL12p70 secretion in the AHI-24 group was comparable with the HIV negative group, indicating a preserved synergistic effect of combined TLR8 and RLR stimulation. However, in the AHI-156 and CHI groups, IL-12p70 production upon co-stimulation was significantly diminished compared to CG (Fig. 3A).

Interestingly, when comparing induction following co-stimulation with the TLR8 and RLR agonist within the groups, the induction of IL-12p70 was on average two-fold in the CG. This induction seemed to be initially preserved in both AHI-groups, even though the absolute cytokine levels were lower. Notably, co-stimulation of monocytes with TLR8 and RLR in the CHI-group led to a decreased induction of IL-12p70 (Fig. S1A).

Stimulation with the RLR agonist alone induced IL-27 production in all groups but the IL-27 expression was lower in the AHI-156 and CHI groups compared to CG. The AHI-24 group had a non-significant lower response compared to HIV negative individuals. The TLR8 agonist did not induce IL-27 (Fig. 3B). Combined stimulation with TLR8 and RLR agonists significantly increased IL-27 production in all groups. The level of IL-27 was comparable between AHI-24 group and CG. In contrast, the AHI-156 and CHI groups exhibited significantly reduced IL-27 levels upon combined stimulation (Fig. 3B).

Taken together, these data suggest that monocytes from individuals who initiated ART during AHI initially retain the ability to produce IL-12p70 and IL-27 upon TLR8 and RLR stimulation. However, this capacity diminishes over time and by 156 weeks post-treatment initiation, their responses become comparable to those observed in CHI individuals. These data indicate that prolonged HIV-1 infection, even with ART initiated in the acute phase of infection, leads to impaired monocyte function and reduced production of key antiviral cytokines.

As expected, induction within the groups following co-stimulation varied as well, with induction of IL-27 being 2.24 fold in the CG group. In AHI-24 this induction was similar, yet seemed to decrease in both the AHI-156 and CHI groups (Fig. S1B). Statistical analysis was performed using unpaired two-way ANOVA with Tukey's post-hoc correction. Significance is indicated as ∗p < 0.05, ∗∗p < 0.01, ∗∗∗p < 0.001, ∗∗∗∗p < 0.0001.

### IL-6 production induced by TLR8 and RLR is altered following infection by HIV

We then evaluated the production of IL-6, a pro-inflammatory cytokine, in response to TLR8 stimulation alone and in combination with RLR stimulation. IL-6 plays a multifaceted role in immune responses and its regulation is important for maintaining a balance between inflammation and antiviral immunity^31^

In line with our previous study,^15^ stimulation with the RLR agonist alone did not induce IL-6 production. Stimulation with the TLR8 agonist alone induced IL-6 production in all groups. Notably, the production of IL-6 in the CHI group was higher compared to CG, and there seemed to be a gradual increase in IL-6 induction in AHI at 24 and 156 weeks compared to CG (Fig. 3C). Following LPS stimulation, IL-6 was induced in all groups, however, while induction between AHI- and CHI-groups was similar, in the CG LPS induced lower levels of IL-6 (Fig. S4A).

We showed earlier that the concerted action between TLR8 and RLR restricts IL-6 production.^15^ As expected, in the CG group, combined TLR8 and RLR stimulation led to a near 2 fold reduction (p < 0.0001) in IL-6 levels compared to TLR8 stimulation alone. The IL-6 restriction in the AHI-24 group was less compared to CG, showing an 1.42 fold increase, however, this proved not to be significant. This reduction in restriction proved highly significant in the other two groups, were we observed a 2.24 and 2.49 fold increase in the AHI-156 and CHI group compared to CG respectively (both p < 0.0001) (Fig. 3C).

Lastly, when comparing the IL-6 restriction observed following co-stimulation with TLR8 and RLR agonists, we observed that the initial IL-6 restriction in the CG group was also observed in the AHI-24 group, but diminished in the AHI-156 and CHI groups, with AHI-156 and CHI being nearly comparable (Fig. 3C, Fig. S1C). Statistical analysis was performed using unpaired two-way ANOVA with Tukey's post-hoc correction. Significance is indicated as ∗p < 0.05, ∗∗p < 0.01, ∗∗∗p < 0.001, ∗∗∗∗p < 0.0001.

These data suggest that in chronically treated HIV infection the restrictive effect of combined TLR8 and RLR activation on IL-6 production is diminished and comparable to AHI-156.

### Crosstalk progressively diminishes over time while inflammatory profile at 24 and 156 weeks remain similar

**Fig. 4:**
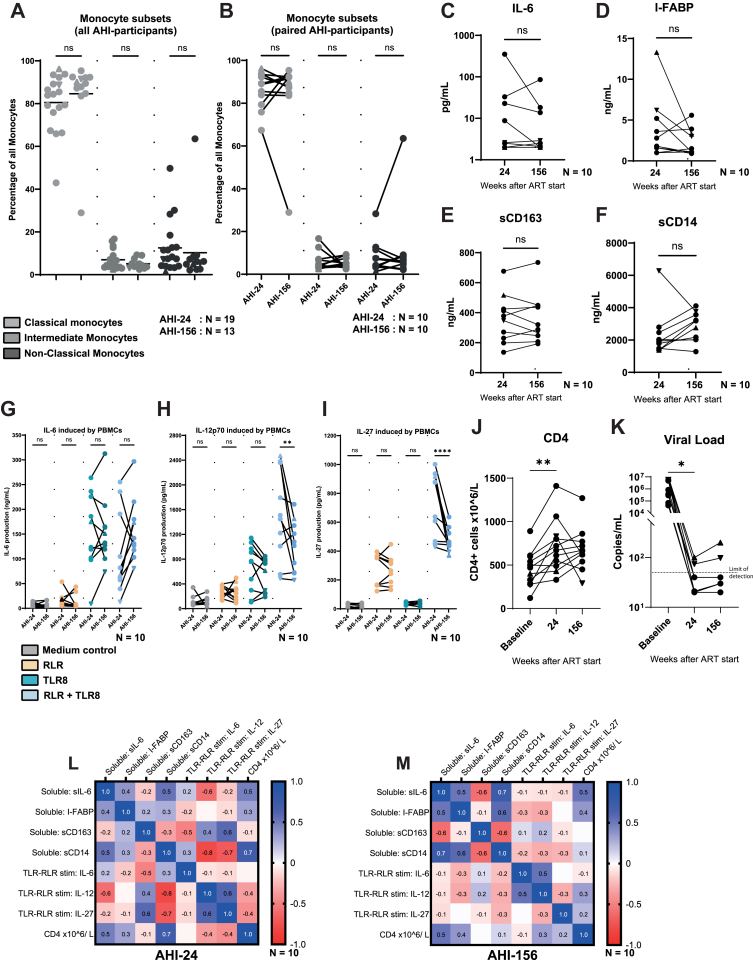
**Responses to TLR8-RLR agonists, but not immunophenotyping, within AHI-donors show decreases in functionality following prolonged infection with HIV**. Monocytes subset were determined based on CD14 and CD16 expression, classical monocytes (CD14++/CD16−), intermediate monocytes (CD14++, CD16+) and non-classical monocytes (CD14+, CD16++). (A) Subpopulations as percentage of total monocytes from all AHI-participants included in the study. AHI-24 n = 19, AHI-156 n = 13. Three biological replicates were used per datapoint. (B) Subpopulations as percentage of total monocytes from the 10 AHI-participants included at the 24 week and 156 week timepoint. Statistical analysis was performed using one-way ANOVA with Tukey's post-hoc correction. AHI-24 n = 10, AHI-156 n = 10. Three biological replicates were used per datapoint. (C–F) Immunophenotyping of soluble IL-6, I-FABP, CD163 and CD14 measured in plasma or serum of paired AHI-participants at both 24 week and 156 week timepoint. Statistical analysis was performed using a paired student T-test. AHI-24 n = 10, AHI-156 n = 10. One biological replicate was used per datapoint. (G–I) Paired analysis of functional immune responses in PBMCs to TLR8 and RLR agonists from paired AHI-participants at both 24 and 156 weeks. Statistical analysis was performed using unpaired two-way ANOVA with Tukey's post-hoc correction. AHI-24 n = 10, AHI-156 n = 10. Three biological replicates were used per datapoint. (J–K) Paired analysis of CD4 counts and viral load in paired AHI-participants at study inclusion (baseline), 24 and 156 weeks. Statistical analysis was performed using a paired student T-test. One biological replicate was used per datapoint. (L–M) Correlation analysis between measured HIV-reservoir, soluble pro-inflammatory factors and measured cytokines following cytokine stimulation. AHI-24 n = 10, AHI-156 n = 10. Significance is indicated as ∗p < 0.05, ∗∗p < 0.01, ∗∗∗p < 0.001, ∗∗∗∗p < 0.0001.

We evaluated monocyte immunophenotypes in the AHI group at 24 weeks and 156 weeks post-ART initiation. The distribution of classical (CD14^high^CD16^neg^), intermediate (CD14^high^CD16^pos^), and non-classical monocyte (CD14^low^CD16^pos^) subsets was comparable between these two timepoints (Fig. 4A, Fig. S3). The subset proportions remained unchanged also when analysis was restricted to the ten participants who provided samples at both 24 and 156 weeks (Fig. 4B). Similarly, soluble markers, measured in serum or plasma, associated with monocyte activation and gut integrity showed no differences over time. Neither Plasma IL-6, I-FABP nor soluble CD163 and CD14 levels differed significantly between 24 and 156 weeks, indicating that systemic inflammation and gut-mucosal damage markers remained stable after the initial phase of ART (Fig. 4C–F).

Monocyte functional responses to TLR8 and RLR agonists were then assessed in paired samples of the AHI-group. Consistent with the preserved immunophenotype, we observed no significant differences between AHI-24 and AHI-156 in baseline (medium control) or single-agonist stimulation conditions (Fig. 4G–I). However, upon combined TLR8 + RLR co-stimulation, a clear reduction in antiviral cytokine induction was evident over time. IL-12p70 and IL-27 production were significantly lower at 156 weeks compared to 24 weeks in the co-stimulation condition (Fig. 4H and I), indicating a loss of TLR8–RLR crosstalk efficacy as ART duration prolonged.

Notably, ART effectively suppressed viral replication and restored CD4+ T cell counts. CD4+ T cell counts increased significantly from baseline to 24 weeks post-ART initiation (Fig. 4J). Correspondingly, plasma viral loads decreased from baseline levels to below the limit of detection in most participants at both 24 and 156 weeks (Fig. 4K). The two AHI participants who experienced low-level viraemia at these timepoints (viral blips of 74 → 98 copies/mL in one donor (indicated by a ▲) and 200 → 100 copies/mL in another (indicated by a ▼)) did not behave as outliers in their cytokine responses, suggesting that minor viral rebounds had no discernible impact on the group's functional profile. Finally, we performed correlation analyses to explore links between various immune parameters at 24 versus 156 weeks. While a few individual correlations reached significance at one or the other timepoint, no consistent pattern emerged between plasma biomarker levels, monocyte cytokine outputs or CD4 counts (Fig. 4L, M). Statistical analysis was performed using unpaired two-way ANOVA with Tukey's post-hoc correction.

In summary, monocyte phenotype and basal inflammation markers remained steady from 24 to 156 weeks, but the synergistic TLR8–RLR “crosstalk” response deteriorated with time on ART, approaching the blunted functional profile observed in chronically treated individuals.

## Discussion

Several studies showed that initiation of ART in acute infection is beneficial in terms of early reduction of inflammation and preservation of innate responses. We showed here that the timing of ART initiation, during acute as well as chronic infection, affects monocyte activation and cytokine responses in PWH. Our study indicates that early ART initiation preserves monocyte function following TLR8 and RLR crosstalk compared to initiating ART during chronic infection. However, this benefit diminishes over time, ultimately aligning with the monocyte function observed in individuals who began treatment during the chronic phase.

PWH who initiate ART during CHI often experience persistent immune activation and incomplete immune restoration, despite effective viral suppression.^32^, ^33^, ^34^ In contrast, individuals who initiate ART during AHI exhibit better immune restoration, with more rapid recovery of CD4+ T cell counts and reduced levels of immune activation markers.^35^, ^36^, ^37^ In our study we investigated specifically monocyte functionality following TLR8 and RLR crosstalk, which reflects antiviral capability. We observed that monocytes from individuals treated during CHI exhibited a heightened activation profile compared to CG, characterised by increased expression of surface markers such as CD11b, CD64, CD163, and HLA-DR. These markers are associated with monocyte activation, phagocytic capacity, antigen presentation, and tissue repair processes.^8^^,^^38^ Two of our monocyte markers, CD11b and CD64, were also elevated at both AHI timepoints. Interestingly, CD11b expression differed from other monocyte activation markers (CD64, CD163, HLA-DR), being significantly elevated in AHI-groups but not in CHI. This differential expression pattern might reflect distinct roles of CD11b in leucocyte migration. The elevated expression of CD11b and CD64 suggests persistent leucocyte migration and phagocytic activity. Such elevation has been described in other HIV-cohorts.^39^ As the immune system reconstitutes following initiation of ART, we observe a normalisation of CD11b expression in the AHI-156 group compared to AHI-24. Increased levels of scavenger receptor CD163 were only observed in the CHI group. As CD163 is associated with activation and anti-inflammatory function the increased expression could reflect inflammation. Similarly, elevated HLA-DR expression may indicate sustained antigen presentation and immune activation.^40^, ^41^, ^42^, ^43^, ^44^

These findings align with previous studies that have reported differences in monocyte activation between acute and chronic HIV infection, where monocytes in CHI exhibit an activated phenotype and produce higher levels of pro-inflammatory cytokines like IL-6.^45^, ^46^, ^47^, ^48^

Gene expression analysis further supports the notion of a different profile of monocyte activation between the three groups. Monocytes from the CHI group showed significant upregulation of multiple pro-inflammatory and interferon-stimulated genes (ISGs), including Mx2, ISG15, CCR2, OAS1, and CXCL10, indicating ongoing immune activation and inflammation despite ART.^23^^,^^25^ In contrast, the AHI-24 group exhibited upregulation of inflammatory markers such as TNF-α and pro-IL-1β, reflecting general inflammation associated with recent infection. In AHI-156, the expression levels of these inflammatory markers decreased to levels comparable to CG, suggesting a normalisation of immune activation over time with sustained ART-induced suppressed viral load. This pattern suggests that early ART initiation facilitates initial immune reconstitution and reduces inflammation but does not prevent immune dysregulation in the long term, as monocyte responses in AHI-156 align with those observed in CHI.^49^^,^^50^

Both TLR8 and RLR agonists are recognised for inducing innate immune responses through their respective receptors. TLR8 detects single-stranded RNA within endosomes, activating signalling pathways that drive the production of pro-inflammatory cytokines and type I interferons.^10^ RLRs, such as RIG-I, detect viral RNA in the cytosol and initiate antiviral responses.^18^ The crosstalk between TLR8 and RLR pathways enhances monocyte activation by synergistically inducing antiviral cytokines Il-12p70 and IL-27, while downregulating pro-inflammatory cytokines like IL-6.^15^ HIV-1 proteins systematically target the ISGF3 complex (STAT1-STAT2-IRF9) through Vif-mediated STAT1/STAT3 degradation and disrupted STAT1 phosphorylation, which is particularly critical for IL-27 induction as an interferon-regulated cytokine.^51^ HIV-1 proteins, such as Nef or VPU, inhibit NFκB,^52^^,^^53^ with Nef especially known to interfere IL-12p40 transcription through the JNK pathway. Furthermore, HIV-1 protease directly target RIG-I,^54^ inhibiting signalling at the source. As these post-translational modifications accumulate over time, early ART initiation may preserve monocyte function only initially.

IL-12p70 and IL-27 are essential for the differentiation and function of Th1 cells and cytotoxic T lymphocytes (CTLs).^55^^,^^56^ These cytokines promote antiviral responses by enhancing the activity of NK-cells and CTLs, and by facilitating the development of protective T cell-mediated immunity.^57^^,^^58^ Functionally, we observed that co-stimulation of TLR8 and RLRs in monocytes from CG led to robust induction of IL-12p70 and IL-27 and a restriction of IL-6. Co-stimulation of monocytes from the AHI-24 group induced IL-12p70 and IL-27 in a similar fashion as in CG, indicating intact immune crosstalk and potential for effective antiviral immune responses. However, in the AHI-156 and CHI groups, the production of IL-12p70 and IL-27 upon TLR8 and RLR co-stimulation was significantly diminished. This suggests that over time, even in individuals who initiated ART during AHI, monocyte function declines, leading to impaired cytokine responses. The reduced production of IL-12p70 and IL-27 can have significant implications for CTL function, as these cytokines are critical for promoting CTL differentiation and enhancing their cytotoxic activity.^59^^,^^60^ The reduction in IL-27 induction can indicate multiple effects too, as IL-27 has pleiotropic effects and is known as a key regulator of immune responses, able to induce follicular T-cells via IL-21 and IL-10 producing Type 1 regulatory T-cells.^61^, ^62^, ^63^

Our observation of elevated IL-6 levels following TLR8 stimulation alone is interesting, as prolonged HIV-infection appears to enhance the potency of IL-6 induction specifically through this pathway. Increases in plasma IL-6 following HIV infection have been documented.^48^^,^^64^ Similarly, following stimulation, monocytes from PWH showed higher potency to induce IL-6.^65^^,^^66^ Elevated IL-6 can negatively impact CTL function by promoting the expression of inhibitory receptors and altering metabolic pathways critical for CTL activity.^67^^,^^68^ Restriction in IL-6 induction following crosstalk between TLR8 and RLR suggests balancing cytokine secretion as displayed by the induction of IL-12p70, IL-27 and the restriction of IL-6. The loss of this ability, as shown in CHI, suggests a reduced ability to shape immunity. This can contribute to immune dysregulation by interfering with T-helper differentiation, impairing effective antiviral immunity.^69^ Interestingly, this pattern of elevated IL-6 and reduced IL-12p70 production in CHI and AHI-156 groups was not limited to TLR8-RLR stimulation but was also observed following LPS stimulation. This suggests a broader dysfunction in monocyte cytokine responses that extends beyond specific PRR pathways, potentially reflecting a fundamental reprogramming of monocyte inflammatory responses in chronic HIV infection despite ART, however, we must acknowledge that these findings are observational in nature and serve as hypothesis generating for future research. Upcoming studies should delve deeper into the mechanistic nature of PRR crosstalk in the context of HIV.

Our observations suggest that despite early ART initiation during AHI, alterations in monocyte expression, transcriptional activity, signalling and function emerge over time, leading to cytokine responses similar to those observed in individuals who initiated ART during CHI. In conclusion, our study demonstrates that monocyte activation and function differ between individuals who initiate ART during AHI and those who are on ART during CHI. Early ART initiation preserves monocyte function and cytokine responses in the short term, but alterations in immune function emerge over time, leading to profiles similar to those observed in treated chronic infection. The observed changes in monocyte cytokine production in the AHI-group between 24 and 156 weeks after treatment initiation highlight the complex interplay between HIV infection and the immune system.

A limitation of our analysis is that the median ART duration between our AHI-156 and CHI group differed significantly (36 months for AHI-156, compared to 24 months in CHI). Both groups have been on ART for a prolonged time, allowing for a measure of generalisation when comparing responses from CHI and AHI-156. While their ART duration differs, the participants demographics between AHI and CHI groups is quite homogeneous, allowing for comparison between groups. Given previously described impact of ethnicity, sex and viral clade on immunological outcomes, our data warrant further validation in cohorts with different backgrounds.^70^ The sample size is modest but sufficient for statistical analyses as the differences between responses are quite robust. We did not have data on anti-CMV antibodies, which could potentially influence monocyte activation levels between individuals. However, we did measure multiple systemic inflammation markers in the AHI participants, and observed no differences between 24 and 156 weeks, nor in baseline cytokine levels in unstimulated cells.

It is noteworthy that classical markers of microbial translocation and monocyte activation in plasma or serum remained stable between 24 and 156 weeks post-ART. In our cohort, plasma IL-6 levels and markers such as I-FABP, sCD163, and sCD14 did not significantly differ between the two timepoints, indicating. These data suggest that the decline in monocyte function at 3 years is not per se associated with an increase in systemic inflammation. Moreover, we found no clear correlations between CD4-counts and monocyte immune parameters at either 24 or 156 weeks. This lack of a consistent relationship implies that the progressive decline in monocyte TLR8–RLR crosstalk function over time is a functional defect rather than a steady-state one. Additionally, other innate immune functions could be negatively affected, including ADCC.

The transient preservation of monocyte function observed in our study has important implications for the timing of immunomodulatory interventions in HIV cure strategies. Our findings identify a narrow therapeutic window post-ART initiation during which monocyte functionality remains intact, suggesting that immunotherapeutic interventions should be initiated as early as possible after diagnosis, and starting ART, to capitalise on this period of preserved innate immune competence. When designing clinical trials for immune-based interventions, researchers should consider that even in early-treated individuals, timepoints later during infection may functionally resemble chronic HIV infection rather than preserved immunity, highlighting the critical importance of intervention timing in HIV cure research. Understanding the mechanisms driving these changes despite ART is crucial for developing strategies to restore immune homoeostasis and improve long-term clinical outcomes for PWH.

## Contributors

KV, TG and GB conceived and designed experiments. KV, PvP, TK, SS, KvD, IM and JvH performed the experiments. KV and PvP acquired and analysed data. KV, PvP, NK, TG and GB interpreted, accessed and verified data and contributed to scientific discussion. RvC, CR, JP, GB: design and governance of NOVA study. GB: Implementation, execution and follow-up of the NOVA study. KV, GB, and TG wrote the manuscript with input from all listed authors. TG and GB perceived of the original study idea and was involved in all aspects of the study. TG and GB vouch for the completeness and accuracy of the data. All authors contributed to the article and approved the submitted version.

The NOVA study team contributed by providing access to NOVA study material.

## Data sharing statement

The original contributions presented in the study are included in the article or supplementary materials. Further enquiries can be directed to the corresponding author (T.B.Geijtenbeek@amsterdamumc.nl) and raw data (study protocols and data) will be made available upon request.

## Statement on usage of generative AI

During the preparation of this work the author(s) used MistralAI's model Magistral Small 1.0 and Mistral Small 3.0 in order to correct grammar and sentence structuring. Both models were run locally using LMStudio. The authors have reviewed and confirmed the validity of the text and take full responsibility for the content of the publication.

## Declaration of interests

CR has received institutional funding from Gilead and Viiv separate from this study (as grants, consulting/advisory fees, support for meetings/travel), and declares stock or stock options from Eli Lily and Immunocore. TBH has received additional funding from ZonMW separate from this study.

The other authors have no conflicts of interest to declare.

## Appendix A Supplementary data

Supplemental Tables

Antibody data sheet

NOVA board team

Supplemental Figures Plus Legends
