# Supplementary material for: Treatment in acute HIV infection only temporarily preserves monocyte function: a comparative cohort study in adult males
Source: eBioMedicine. 2025 Nov 7;122:105997. doi: 10.1016/j.ebiom.2025.105997 (PMC12790590; doi:10.1016/j.ebiom.2025.105997)
Supplement: Supplemental Tables [file mmc1.docx]

**Table S1 Function of monocyte cell surface markers**

| **Surface marker** | **Other nomenclature** | **Function** |
| --- | --- | --- |
| **CD11b** | Integrin alpha M (ITGAM) | CD11b is essential for cell adhesion and migration. It is involved in recognizing and binding to complement-coated pathogens and phagocytosis. |
| **CD64** | Fc gamma receptor I (FcγRI) | High-affinity receptor for the Fc region of IgG and involved in antibody-dependent cellular phagocytosis. |
| **CD163** | Hemoglobin Scavenger Receptor | Scavenging receptor that plays a role in clearing hemoglobin/haptoglobin complexes. |
| **HLA-DR** |  | Class II MHC molecule involved in presenting peptides to CD4+ T cells. It plays a key role in initiating immune responses by helping T cells recognize foreign antigens. HLA-DR molecules are highly polymorphic, which increases the variety of immune responses within the population​​. |

**Table S2 mRNA transcripts investigated and their functions**

| **mRNA transcript** | | **Function** |
| --- | --- | --- |
| **Mx2** | Myxovirus resistance protein 2 | Interferon-induced protein that blocks HIV-1 nuclear import and inhibits viral replication. |
| **CCR2** | C-Chemokine Receptor type 2 | Receptor that recruits monocytes to inflammation sites, contributing to chronic inflammation in HIV. |
| **CXCL10** | C-X-C motif chemokine 10 | Interferon-induced protein involved in attracting T and NK cells to infection sites, playing a key role in antiviral immune responses. |
| **ISG15** | Interferon Stimulated Gene 15 | Interferon-induced protein that modifies target proteins, enhancing antiviral defense and inhibiting viral replication. |
| **MxA** | Myxovirus resistance protein A | Interferon-stimulated protein that inhibits viral replication, primarily targeting RNA viruses. |
| **APOBEC3G** | Apolipoprotein B mRNA-editing enzyme catalytic polypeptide-like 3G | A cytidine deaminase that mutates the HIV genome during reverse transcription, though counteracted by HIV Vif protein. |
| **BST2** | Bone marrow stromal antigen 2 | Prevents HIV-1 virion release from infected cells, limiting viral budding, though counteracted by HIV Vpu. |
| **OAS1** | 2'-5'-Oligoadenylate synthetase 1 | Activates RNase to degrade viral RNA, contributing to the antiviral response. |
| **ADAR** | Adenosine deaminase acting on RNA | Edits RNA by converting adenosine to inosine, thereby altering viral replication and immune responses. |
| **TNFα** | Tumor Necrosis Factor α | A key pro-inflammatory cytokine driving immune responses and inflammation. |
| **Pro-IL-1β** | Pro-Interleukin 1 β | Inactive precursor of IL-1β, a major pro-inflammatory cytokine involved in infection response. |
| **SOCS1** | Suppressor of Cytokine Signalling 1 | A negative regulator of cytokine signaling, preventing excessive immune activation and inflammation. |

**Table S3 Benjamini-Hochberg FDR correction for p-values displayed in figure 2.**

| **Rank** | **Comparison**  **(Cytokine, groupA vs groupB)** | **Unadjusted**  **p-value** | **Critical value**  **(**$\frac{\boldsymbol{i}}{\boldsymbol{m}}$ **x Q)** | **Adjusted p-value**  **(q-value = min(**$\frac{\boldsymbol{p x m}}{\boldsymbol{i}}$**)** | **FDR significant?** |
| --- | --- | --- | --- | --- | --- |
| 1 | ADAR, CG – AHI24 | p < 0.0001 | 0.000694 | p = 0.0024 | Yes |
| 2 | ADAR, AHI24 – CHI | p < 0.0001 | 0.001389 | p = 0.0024 | Yes |
| 3 | SOCS1, CG – AHI24 | p < 0.0001 | 0.002083 | p = 0.0024 | Yes |
| 4 | SOCS1, AHI24 – CHI | p = 0.0002 | 0.002778 | p = 0.0036 | Yes |
| 5 | ADAR, CG – AHI156 | p = 0.0023 | 0.003472 | p = 0.0331 | Yes |
| 6 | Mx2, AHI24 – CHI | p = 0.0032 | 0.004167 | p = 0.0368 | Yes |
| 7 | Pro-IL-1β, CG – AHI24 | p = 0.0042 | 0.004861 | p = 0.0368 | Yes |
| 8 | Pro-IL-1β, AHI24 – CHI | p = 0.0042 | 0.005556 | p = 0.0368 | Yes |
| 9 | CCR2, CG – CHI | p = 0.0046 | 0.006250 | p = 0.0368 | Yes |
| 10 | CCR2, AHI156 – CHI | p = 0.0072 | 0.006944 | p = 0.0518 | No |
| 11 | TNFα, CG – AHI24 | p = 0.0267 | 0.007639 | p = 0.1749 | No |
| 12 | CCR2, AHI24 – CHI | p = 0.0361 | 0.008333 | p = 0.2166 | No |
| 13 | Mx2, CG – CHI | p = 0.0391 | 0.009028 | p = 0.2166 | No |

Significant (p > 0.05) values from figure 2 were subjected to Benjamini-Hochbergs FDR correction. Critical values were determined using the formula ($\frac{i}{m}$ x 0.05), with i = rank, m = total number of tests and Q as FDR threshold, i was determined by unadjusted p-value, m set at 72 (6 individual tests for 12 different cytokines. Q was set at 0,05. Subsequently, adjusted p-values (q-values) were calculated using the formula q = min($\frac{p x m}{i}$).
