# Supplementary material for: Treatment in acute HIV infection only temporarily preserves monocyte function: a comparative cohort study in adult males
Source: eBioMedicine. 2025 Nov 7;122:105997. doi: 10.1016/j.ebiom.2025.105997 (PMC12790590; doi:10.1016/j.ebiom.2025.105997)
Supplement: Antibody data sheet [file mmc2.pdf]

Alexa Fluor® 700 anti-human CD11b Antibody

**Catalog# / Size**	301355 / 25 tests<br>301356 / 100 tests
**Clone**	ICRF44
**Regulatory Status**	RUO
**Workshop**	IV M047
**Other Names**	Integrin αM chain, C3biR, CR3, Mac-1, Mo1, ITGAM
**Isotype**	Mouse IgG1, κ
**Description**	CD11b is a 165-170 kD type I transmembrane glycoprotein also known as α_M integrin, Mac-1, CR3, and C3biR. CD11b non-covalently associates with integrin β_2 (CD18) and is expressed on granulocytes, monocytes/macrophages, dendritic cells, NK cells, and subsets of T and B cells. CD11b/CD18 is critical for the transendothelial migration of monocytes and neutrophils. It is also involved in granulocyte adhesion, phagocytosis, and neutrophil activation. CD11b/CD18 interacts with ICAM-1 (CD54), ICAM-2 (CD102), ICAM-4, CD14, CD23, heparin, iC3b, fibrinogen, and factor X.

Product Details

**Verified Reactivity**	Human, Cynomolgus, Rhesus
**Reported Reactivity**	African Green, Baboon, Chimpanzee, Common Marmoset, Pig
**Antibody Type**	Monoclonal
**Host Species**	Mouse
**Formulation**	Phosphate-buffered solution, pH 7.2, containing 0.09% sodium azide and BSA (origin USA)
**Preparation**	The antibody was purified by affinity chromatography and conjugated with Alexa Fluor® 700 under optimal conditions.
**Concentration**	Lot-specific (to obtain lot-specific concentration and expiration, please enter the lot number in our Certificate of Analysis online tool.)
**Storage & Handling**	The antibody solution should be stored undiluted between 2°C and 8°C, and protected from prolonged exposure to light. **Do not freeze.**
**Application**	FC - *Quality tested*
**Recommended Usage**	Each lot of this antibody is quality control tested by immunofluorescent staining with flow cytometric analysis. For flow cytometric staining, the suggested use of this reagent is 5 µl per million cells in 100 µl staining volume or 5 µl per 100 µl of whole blood.<br><br>* Alexa Fluor® 700 has a maximum emission of 719 nm when it is excited at 633 nm / 635 nm. Prior to using Alexa Fluor® 700 conjugate for flow cytometric analysis, please verify your flow cytometer's capability of exciting and detecting the fluorochrome.<br><br>Alexa Fluor® and Pacific Blue™ are trademarks of Life Technologies Corporation.<br><br>View full statement regarding label licenses
**Excitation Laser**	Red Laser (633 nm)
**Application Notes**	The ICRF44 antibody inhibits heterotypic adhesion of granulocytes in response to fMLP. Additional reported applications (for the relevant formats) include: immunohistochemical staining of acetone-fixed frozen tissue sections, immunofluorescence microscopy[5], stimulation of monocytes[3], blocking of heterotypic PMN aggregation[8], and blocking of granulocyte activation[12]. This clone was tested in-house and does not work on formalin fixed paraffin-embedded (FFPE) tissue.

The Ultra-LEAF™ purified antibody (Endotoxin < 0.01 EU/µg, Azide-Free, 0.2 µm filtered) is recommended for functional assays (Cat. Nos. 301361 & 301362).

Application References

1. Knapp W. 1989. Leucocyte Typing IV. Oxford University Press New York.
2. Barclay N, *et al.* 1997. The Leucocyte Antigen Facts Book. Academic Press Inc. San Diego.
3. Rezzonico R, *et al.* 2001. *Blood* 97:2932. (Stim)
4. Marsik C, *et al.* 2003. *Shock* 20:493. (FC)
5. David A, *et al.* 2003. *J. Leukoc. Biol.* 74:551. (IF)
6. Charles N, *et al.* 2010. *Nat. Med.* 16:701. (FC) PubMed
7. Thurlow LR, *et al.* 2010. *Infect. Immun.* 128:1128. (FC) PubMed
8. Jadhav S, *et al.* 2001. *J. Immunol.* 167:5986. (Block)
9. Yoshino N, *et al.* 2000. *Exp. Anim. (Tokyo)* 49:97. (FC)
10. Sestak K, *et al.* 2007. *Vet. Immunol. Immunopathol.* 119:21. (FC)
11. Wen T, *et al.* 2014. *J Immunol.* 192:5481. (FC) PubMed
12. Sprong T, *et al.* 2003. *Blood* 102:3702. (Block)

See More

Product Citations

1. Gao R, *et al.* 2021. iScience. 24:103133. PubMed
2. Bettini E, *et al.* 2022. STAR Protoc. 3:101840. PubMed
3. Aru B, *et al.* 2023. Balkan Med J. 40:117. PubMed
4. Houlder E, *et al.* 2023. Nat Commun. 14:1863. PubMed
5. Wiesner DL, *et al.* 2020. Cell Host Microbe. 614:27. PubMed
6. Lo MW, *et al.* 2022. Clin Transl Immunology. 11:e1413. PubMed
7. Hakki S, *et al.* 2022. Sci Rep. 12:1427. PubMed
8. Lederer K, *et al.* 2022. Cell. . PubMed
9. Hunegnaw R, *et al.* 2019. Front Immunol. 1.484027778. PubMed

RRID

AB_2750074 (BioLegend Cat. No. 301355)
AB_2750075 (BioLegend Cat. No. 301356)

Antigen Details

Structure Integrin, type I transmembrane glycoprotein, associates with integrin β_2 (CD18), 165-170 kD

Distribution Granulocytes, monocytes/macrophages, dendritic cells, NK cells, subset of T cells, subset of B cells

Function Adhesion, phagocytosis, chemotaxis, neutrophil activation

Ligand/Receptor ICAM-1(CD54), ICAM-2 (CD102), ICAM-4, CD14, CD23, heparin, iC3b, fibrinogen, factor X

Cell Type B cells, Dendritic cells, Granulocytes, Macrophages, Monocytes, Neutrophils, NK cells, T cells, Tregs

Biology Area Cell Adhesion, Cell Biology, Costimulatory Molecules, Immunology, Innate Immunity, Neuroscience, Neuroscience Cell Markers

Molecular Family Adhesion Molecules, CD Molecules

Antigen References 1. Stewart M, *et al.* 1995. *Curr Opin Cell Biol.* 7:690.

Gene ID 3684

Related Protocols

- Cell Surface Flow Cytometry Staining Protocol

Other Formats

APC anti-human CD11b, Biotin anti-human CD11b, PE anti-human CD11b, PE/Cyanine5 anti-human CD11b, Purified anti-human CD11b, Pacific Blue™ anti-human CD11b, Alexa Fluor® 488 anti-human CD11b, Alexa Fluor® 647 anti-human CD11b, PE/Cyanine7 anti-human CD11b, PerCP/Cyanine5.5 anti-human CD11b, Brilliant Violet 421™ anti-human CD11b, Brilliant Violet 570™ anti-human CD11b, FITC anti-human CD11b, Brilliant Violet 605™ anti-human CD11b, Brilliant Violet 510™ anti-human CD11b, Brilliant Violet 650™ anti-human CD11b, Purified anti-human CD11b (Maxpar® Ready), Alexa Fluor® 594 anti-human

CD11b, APC/Cyanine7 anti-human CD11b, Brilliant Violet 711™ anti-human CD11b, Brilliant Violet 785™ anti-human CD11b, PE/Dazzle™ 594 anti-human CD11b, APC/Fire™ 750 anti-human CD11b, TotalSeq™-A0161 anti-human CD11b, Alexa Fluor® 700 anti-human CD11b, TotalSeq™-B0161 anti-human CD11b, TotalSeq™-C0161 anti-human CD11b, Ultra-LEAF™ Purified anti-human CD11b, TotalSeq™-D0161 anti-human CD11b, GMP PE/Cyanine7 anti-human CD11b, GMP PE anti-human CD11b, Spark UV™ 387 anti-human CD11b, GMP PerCP/Cyanine5.5 anti-human CD11b, GMP APC anti-human CD11b, GMP FITC anti-human CD11b, GMP APC/Fire™ 750 anti-human CD11b, Spark Blue™ 515 anti-human CD11b, Spark Violet™ 500 anti-human CD11b, Spark Red™ 718 anti-human CD11b (Flexi-Fluor™), GMP Pacific Blue™ anti-human CD11b, APC/Fire™ 810 anti-human CD11b, Brilliant Violet 750™ anti-human CD11b Antibody

Product Data

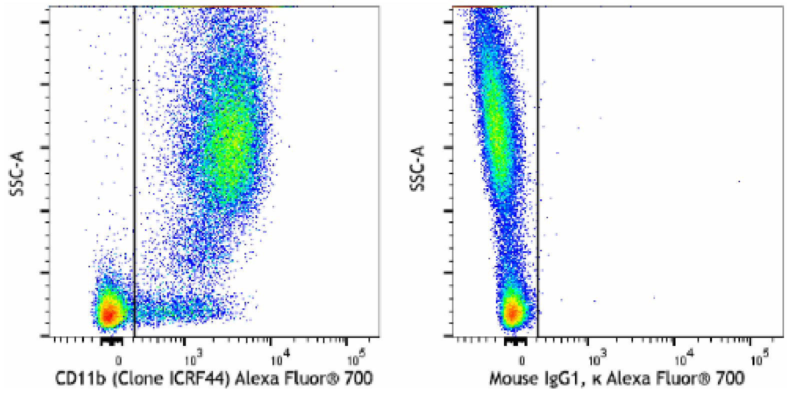

Human peripheral blood lymphocytes, monocytes, and granulocytes were stained with CD11b (clone ICRF44) Alexa Fluor® 700 (left) or mouse IgG1, κ Alexa Fluor® 700 isotype control (right).

For Research Use Only. Not for diagnostic or therapeutic use.

This product is supplied subject to the terms and conditions, including the limited license, located at www.biolegend.com/terms) ("Terms") and may be used only as provided in the Terms. Without limiting the foregoing, BioLegend products may not be used for any Commercial Purpose as defined in the Terms, resold in any form, used in manufacturing, or reverse engineered, sequenced, or otherwise studied or used to learn its design or composition without express written approval of BioLegend. Regardless of the information given in this document, user is solely responsible for determining any license requirements necessary for user's intended use and assumes all risk and liability arising from use of the product. BioLegend is not responsible for patent infringement or any other risks or liabilities whatsoever resulting from the use of its products.

BioLegend, the BioLegend logo, and all other trademarks are property of BioLegend, Inc. or their respective owners, and all rights are reserved.

8999 BioLegend Way, San Diego, CA 92121 www.biolegend.com
Toll-Free Phone: 1-877-Bio-Legend (246-5343) Phone: (858) 768-5800 Fax: (877) 455-9587

CD16 Monoclonal Antibody (eBioCB16 (CB16)), eFluor™ 450, eBioscience™

Product Details	
Size	100 Tests
Species Reactivity	Human
Published Species	C. elegans, Human
Host/Isotype	Mouse / IgG1, kappa
Recommended Isotype Control	Mouse IgG1 kappa Isotype Control (P3.6.2.8.1), eFluor™ 450, eBioscience™
Class	Monoclonal
Type	Antibody
Clone	eBioCB16 (CB16)
Conjugate	eFluor™ 450
Excitation/Emission Max	405/445 nm
Form	Liquid
Concentration	5 µL/Test
Purification	Affinity chromatography
Storage buffer	PBS, pH 7.2, with BSA
Contains	0.09% sodium azide
Storage conditions	4°C, store in dark, DO NOT FREEZE!
RRID	AB_1272052

Applications	Tested Dilution	Publications
Immunocytochemistry (ICC/IF)	-	2 Publications
Flow Cytometry (Flow)	5 µL (0.25 µg)/test	27 Publications
T-Cell Activation (TCA)	-	1 Publication

Product Specific Information

Description: The eBioCB16 monoclonal antibody recognizes CD16 (Fc gammaRIII), the low-affinity receptor for IgG with an apparent molecular weight of 50-80 kDa. CD16 is represented by two similar genes, CD16A (Fc gammaRIIIA), which exists as a hetero-oligomeric polypeptide-anchored form in macrophages and NK cells and CD16B (Fc gammaRIIIB), which exist as a monomeric GPI-anchored form in neutrophils. Furthermore, there are two known polymorphisms of CD16B, NA-1 and NA-2. Individuals homozygous for NA-2 show a lower phagocytic capacity compared with NA-1. CD16 binds IgG in the form of immune complexes and shows preferential binding of IgG1 and IgG3 isotypes and minimal binding of IgG2 and IgG4. Upon IgG binding, both CD16 isoforms initiate signal transduction cascades that lead to a variety of responses including antibody-dependent cell-mediated cytotoxicity (ADCC), phagocytosis, degranulation and proliferation.

Applications Reported: This eBioCB16 (CB16) antibody has been reported for use in flow cytometric analysis.

Applications Tested: This eBioCB16 (CB16) antibody has been pre-titrated and tested by flow cytometric analysis of normal human peripheral blood cells. This can be used at 5 µL (0.25 µg) per test. A test is defined as the amount (µg) of antibody that will stain a cell sample in a final volume of 100 µL. Cell number should be determined empirically but can range from 10^5 to 10^8 cells/test.

eFluor® 450 is an alternative to Pacific Blue®. eFluor® 450 emits at 445 nm and is excited with the Violet laser (405 nm). Please make sure that your instrument is capable of detecting this fluorochome.

Excitation: 405 nm; Emission: 445 nm; Laser: Violet Laser.

Filtration: 0.2 µm post-manufacturing filtered.

Product Images For CD16 Monoclonal Antibody (eBioCB16 (CB16)), eFluor™ 450, eBioscience™

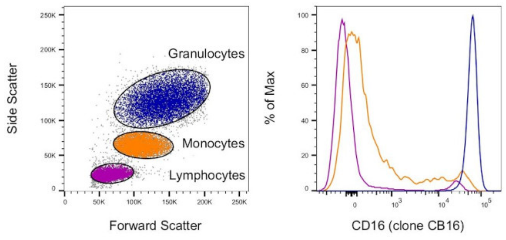

CD16 Antibody (48-0168-42)
Staining of human peripheral blood cells. As expected based on known relative expression patterns, CD16 clone CB16 stains all granulocytes, a subset of monocytes and a subset of lymphocytes (NK cells). Details: Normal human whole blood was surface stained with CD16 (clone CB16). After staining, red blood cells were lysed using 1-step Fix/Lyse Buffer. Cells in the lymphocyte (purple histogram), monocyte (orange histogram), or granulocyte (blue histogram) gates were used for analysis. {RE}

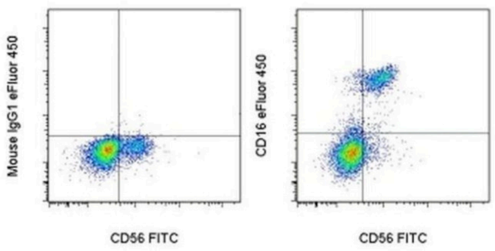

CD16 Antibody (48-0168-42) in Flow
Staining of normal human peripheral blood cells with Anti-Human CD56 (NCAM) FITC (Product # 11-0569) and Mouse IgG1 K Isotype Control eFluor® 450 (Product # 48-4714-82) (left) or Anti-Human CD16 eFluor® 450 (right). Cells in the lymphocyte gate were used for analysis.

View more figures on thermofisher.com

30 References

Immunocytochemistry (2)

OncoTargets and therapy

A DNA vaccine encoding mutated HPV58 mE6E7-Fc-GPI fusion antigen and GM-CSF and B7.1.

"Published figure using CD16 monoclonal antibody (Product # 48-0168-42) in Immunofluorescence"

Authors: Wang H,Yu J,Li L

Year
2015

Arthritis research & therapy

Decreased plasma levels of soluble CD18 link leukocyte infiltration with disease activity in spondyloarthritis.

"Published figure using CD16 monoclonal antibody (Product # 48-0168-42) in Immunofluorescence"

Authors: Kragstrup TW,Jalilian B,Hvid M,Kjærgaard A,Østgård R,Schiøttz-Christensen B,Jurik AG,Robinson WH,Vorup-Jensen T,Deleuran B

Year
2014

Flow Cytometry (27)

Journal of experimental & clinical cancer research : CR

NK cell-triggered CCL5/IFN-CXCL9/10 axis underlies the clinical efficacy of neoadjuvant anti-HER2 antibodies in breast cancer.

"Published figure using CD16 monoclonal antibody (Product # 48-0168-42) in Flow Cytometry"

Authors: Santana-Hernández S,Suarez-Olmos J,Servitja S,Berenguer-Molins P,Costa-Garcia M,Comerma L,Rea A, Perera-Bel J,Menendez S,Arpí O,Bermejo B,Martínez MT,Cejalvo JM,Comino-Méndez I,Pascual J,Alba E,López-Botet M,Rojo F,Rovira A,Albanell J,Muntasell A

Year
2024

PeerJ

SARS-CoV-2 Delta (B.1.617.2) variant replicates and induces syncytia formation in human induced pluripotent stem cell-derived macrophages.

"Published figure using CD16 monoclonal antibody (Product # 48-0168-42) in Flow Cytometry"

Authors: Thaweerattanasinp T,Wanitchang A,Saenboonrueng J,Srisutthisamphan K,Wanasen N,Sungsuwan S, Jongkaewwattana A,Chailangkarn T

Year
2023

View more Flow references on thermofisher.com

More applications with references on thermofisher.com

TCA (1)

For Research Use Only. Not for use in diagnostic procedures. Not for resale without express authorization. Products are warranted to operate or perform substantially in conformance with published Product specifications in effect at the time of sale, as set forth in the Production documentation, specifications and/or accompanying package inserts ("Documentation"). No claim of suitability for use in applications regulated by FDA is made. The warranty provided herein is valid only when used by properly trained individuals. Unless otherwise stated in the Documentation, this warranty is limited to one year from date of shipment when the Product is subjected to normal, proper and intended usage. This warranty does not extend to anyone other than the Buyer. Any model or sample furnished to Buyer is merely illustrative of the general type and quality of goods and does not represent that any Product will conform to such model or sample. NO OTHER WARRANTIES, EXPRESS OR IMPLED, ARE GRANTED INCLUDING WITHOUT LIMITATION, IMPLIED WARRANTIES OF MERCHANTABILITY, FITNESS FOR ANY PARTICULAR PURPOSE, OR NON INFRINGEMENT. BUYER'S EXCLUSIVE REMEDY FOR NON-CONFORMING PRODUCTS DURING THE WARRANTY PERIOD IS LIMITED TO REPAIR, REPLACEMENT OF OR REFUND FOR THE NON-CONFORMING PRODUCT(S) AT SELLER'S SOLE OPTION. THERE IS NO OBLIGATION TO REPAIR, REPLACE OR REFUND FOR PRODUCTS AS THE RESULT OF (I) ACCIDENT, DISASTER OR EVENT OF FORCE MAJEURE, (II) MISUSE, FAULT OR NEGLIGENCE OF OR BY BUYER, (III) USE OF THE PRODUCTS IN A MANNER FOR WHICH THEY WERE NOT DESIGNED, OR (IV) IMPROPER STORAGE AND HANDLING OF THE PRODUCTS. Unless otherwise expressly stated on the Product or in the documentation accompanying the Product, the Product is intended for research only and is not to be used for any other purpose, including without limitation, unauthorized commercial uses, in vitro diagnostic uses, ex vivo or in vivo therapeutic uses, or any type of consumption by or application to human or animals.

APC anti-human CD163 Antibody

Catalog# / Size
326509 / 25 tests
326510 / 100 tests

Clone
RM3/1

Regulatory Status
RUO

Other Names
GHI/61, M130, RM3/1, p155, Hemoglobin/haptoglobin complex receptor, Macrophage-associated antigen, ED2(rat), Macrophage marker

Isotype
Mouse IgG1, κ

Description
CD163 is a member of the group B scavenger receptor cysteine-rich superfamily, also known as GHI/61, M130, RM3/1, p155, hemoglobin-haptoglobin complex receptor, or macrophage-associated antigen. It is a 134 kD (non-reduced)/155 kD (reduced) glycoprotein primarily expressed on macrophages, Kuffer cells, monocytes, subset of dendritic cells, and a subset of hematopoietic stem/progenitor cells. CD163 binds to haptoglobin-hemoglobin complex and TWEAK, and plays a role in clearing hemoglobin and regulating cytokine production by macrophages. Membrane CD163 can be cleaved by metalloproteinases (MMP), resulting in a soluble form. Elevated serum level of sCD163 has been implicated in many kinds of inflammation diseases.

Product Details

Verified Reactivity
Human

Antibody Type
Monoclonal

Host Species
Mouse

Immunogen
Human monocytes

Formulation
Phosphate-buffered solution, pH 7.2, containing 0.09% sodium azide and BSA (origin USA)

Preparation
The antibody was purified by affinity chromatography and conjugated with APC under optimal conditions.

Concentration
Lot-specific (to obtain lot-specific concentration and expiration, please enter the lot number in our Certificate of Analysis online tool.)

Storage & Handling
The antibody solution should be stored undiluted between 2°C and 8°C, and protected from prolonged exposure to light. **Do not freeze.**

Application
FC - Quality tested

Recommended Usage
Each lot of this antibody is quality control tested by immunofluorescent staining with flow cytometric analysis. For flow cytometric staining, the suggested use of this reagent is 5 µl per million cells in 100 µl staining volume or 5 µl per 100 µl of whole blood.

Excitation Laser
Red Laser (633 nm)

Application Notes
Clone RM3/1 binds to domain 9 of CD163. Additional reported applications (for the relevant formats) include: immunofluorescence[7].

Application References

1. Högger P, *et al.* 1998. *J. Immunol.* 161:1883. (FC)
2. Zwadlo G, *et al.* 1987. *Exp. Cell Biol.* 55:295. (FC)
3. Buechler C, *et al.* 2000. *J. Leukoc. Biol.* 67:97. (FC)
4. Puig-Kroger A, *et al.* 2009. *Cancer Res.* 69:9395. (FC) PubMed
5. Madsen M, *et al.* 2004. *J. Biol. Chem.* 279:51561. (FC)
6. Jones K, *et al.* 2013. *Clin Cancer Res.* 19:731. (FC) PubMed
7. Stewart DA, *et al.* 2012. *Mol. Cancer Res.* 10:727. (IF)

Product Citations

1. Al Dulaimi D, *et al.* 2018. Front Immunol. 1052:9. PubMed
2. Lou Y, *et al.* 2023. Reprod Biol Endocrinol. 50:21. PubMed
3. Song M, *et al.* 2023. iScience. 107325:26. PubMed
4. Zhang S, *et al.* 2020. Front Immunol. 1.757638889. PubMed

RRID AB_2564014 (BioLegend Cat. No. 326509)
AB_2564015 (BioLegend Cat. No. 326510)

Antigen Details

Structure 134 kD (non-reduced)/155 kD (reduced) glycoprotein, Scavenger receptor superfamily

Distribution Monocytes, macrophages, Kuffer cells, subset of dendritic cells, subset of hematopoietic stem/progenitor cells

Function Clearance of haptoglobin-hemoglobin complex, regulation of cytokine production by macrophages

Ligand/Receptor Haptoglobin-hemoglobin complex, TWEAK

Cell Type Monocytes, Macrophages, Dendritic cells, Hematopoietic stem and progenitors

Biology Area Cell Biology, Immunology, Neuroscience, Neuroscience Cell Markers

Molecular Family CD Molecules

Antigen References

1. Roth J, *et al.* 1994. *Transolantation.* 57:127.
2. Van den Heuvel MM, *et al.1999. J. Leukoc. Biol. 66:858.*
3. *Sulahian TH, et al. 2000. Cytokines 12:1312.*
4. *Fabriek BO, et al. 2007. J. Neuroimmunol. 187:179.*

Gene ID 9332

Related Protocols

- Cell Surface Flow Cytometry Staining Protocol

Other Formats

Purified anti-human CD163, PE anti-human CD163, Alexa Fluor® 647 anti-human CD163, APC anti-human CD163, PerCP/Cyanine5.5 anti-human CD163, PE/Cyanine7 anti-human CD163

Product Data

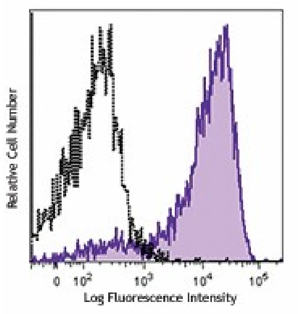

Human peripheral blood monocytes were incubated overnight with IL-10 and then stained with anti-human CD163 (clone RMA3/1) APC (filled histogram) or mouse IgG1, κ APC isotype control (open histogram).

For Research Use Only. Not for diagnostic or therapeutic use.

This product is supplied subject to the terms and conditions, including the limited license, located at www.biolegend.com/terms) ("Terms") and may be used only as provided in the Terms. Without limiting the foregoing, BioLegend products may not be used for any Commercial Purpose as defined in the Terms, resold in any form, used in manufacturing, or reverse engineered, sequenced, or otherwise studied or used to learn its design or composition without express written approval of BioLegend. Regardless of the information given in this document, user is solely responsible for determining any license requirements necessary for user's intended

use and assumes all risk and liability arising from use of the product. BioLegend is not responsible for patent infringement or any other risks or liabilities whatsoever resulting from the use of its products.

BioLegend, the BioLegend logo, and all other trademarks are property of BioLegend, Inc. or their respective owners, and all rights are reserved.

8999 BioLegend Way, San Diego, CA 92121 www.biolegend.com
Toll-Free Phone: 1-877-Bio-Legend (246-5343) Phone: (858) 768-5800 Fax: (877) 455-9587

APC/Cyanine7 anti-human CD16 Antibody

**Catalog# / Size**	302017 / 25 tests<br>302018 / 100 tests
**Clone**	3G8
**Regulatory Status**	RUO
**Workshop**	V NK80
**Other Names**	FcγRIII, Fc gamma receptor, Fc gamma receptor 3
**Isotype**	Mouse IgG1, κ
**Description**	CD16 is known as low affinity IgG receptor III (FcγRIII). It is expressed as two distinct forms (CD16a and CD16b). CD16a (FcγRIIIA) is a 50-65 kD polypeptide-anchored transmembrane protein. It is expressed on the surface of NK cells, activated monocytes, macrophages, and placental trophoblasts in humans. CD16b (FcγRIIIB) is a 48 kD glycosylphosphatidylinositol (GPI)-anchored protein. Its extracellular domain is over 95% homologous to that of CD16a, and it is expressed specifically on neutrophils. CD16 binds aggregated IgG or IgG-antigen complex which functions in NK cell activation, phagocytosis, and antibody-dependent cell-mediated cytotoxicity (ADCC).

Product Details

**Verified Reactivity**	Human, Cynomolgus, Rhesus
**Reported Reactivity**	African Green, Baboon, Capuchin Monkey, Chimpanzee, Common Marmoset, Pigtailed Macaque, Sooty Mangabey, Squirrel Monkey
**Antibody Type**	Monoclonal
**Host Species**	Mouse
**Immunogen**	Human PMN cells
**Formulation**	Phosphate-buffered solution, pH 7.2, containing 0.09% sodium azide and BSA (origin USA)
**Preparation**	The antibody was purified by affinity chromatography, and conjugated with APC/Cyanine7 under optimal conditions.
**Concentration**	Lot-specific (to obtain lot-specific concentration and expiration, please enter the lot number in our Certificate of Analysis online tool.)
**Storage & Handling**	The antibody solution should be stored undiluted between 2°C and 8°C, and protected from prolonged exposure to light. **Do not freeze.**
**Application**	FC - *Quality tested*
**Recommended Usage**	Each lot of this antibody is quality control tested by immunofluorescent staining with flow cytometric analysis. For flow cytometric staining, the suggested use of this reagent is 5 µl per million cells in 100 µl staining volume or 5 µl per 100 µl of whole blood.
**Excitation Laser**	Red Laser (633 nm)
**Application Notes**	The 3G8 antibody clone blocks neutrophil phagocytosis and stimulates NK cell proliferation. It has been reported that this clone interacts with the FcγRIIa and FcγRIIIb receptors causing neutrophil activation and aggregation[18]. Due to this phenomenon staining in whole blood may cause a reduction in the number of granulocytes or alter their scatter profile.<br><br>Additional reported applications (for the relevant formats) include: immunohistochemical staining of acetone-fixed frozen tissue sections[6], immunoprecipitation[3], stimulation of NK cell proliferation[4], blocking of phagocytosis[5], and blocking of immunoglobulin binding to FcγRIII[7,8]. The Ultra-LEAF™

purified antibody (Endotoxin < 0.01 EU/µg, Azide-Free, 0.2 µm filtered) is recommended for functional assays (Cat. No. 302049, 302050, 302057, 302058).

Application References

1. Knapp W, *et al.* Eds. 1989. Leucocyte Typing IV. Oxford University Press. New York.
2. Schlossman S, *et al.* Eds. 1995. Leucocyte Typing V. Oxford University Press. New York.
3. Edberg J, *et al.* 1997. *J. Immunol.* 159:3849. (IP)
4. Hoshino S, *et al.* 1991. *Blood* 78:3232. (Stim)
5. Tamm A, *et al.* 1996. *Immunol.* 157:1576. (Block)
6. Da Silva DM, *et al.* 2001. *Int. Immunol.* 13:633. (IHC)
7. Holl V, *et al.* 2004. *J. Immunol.* 173:6274. (Block)
8. Hober D, *et al.* 2002. *J. Gen. Virol.* 83:2169. (Block)
9. Brainard DM, *et al.* 2009. *J. Virol.* 83:7305. PubMed
10. Smed-Sörensen A, *et al.* 2008. *Blood* 111:5037. (Block) PubMed
11. Timmerman KL, *et al.* 2008. *J. Leukoc. Biol.* 84:1271. (FC) PubMed
12. Yoshino N, *et al.* 2000. *Exp. Anim. (Tokyo)* 49:97. (FC)

See More

Product Citations

1. Gargaro M, *et al.* 2022. Immunity. 55:1032. PubMed
2. Fatehi Hassanabad A, *et al.* 2022. JTCVS Open. 12:118. PubMed
3. Liisborg C, *et al.* 2022. Acta Ophthalmol. :3. PubMed
4. Hastie KM, *et al.* 2023. Cell Rep. 42:112421. PubMed
5. Jundi B, *et al.* 2021. JCI Insight. 6:e148866. PubMed
6. Lee Y, *et al.* 2020. J Innate Immun. 1:. PubMed
7. Lo MW, *et al.* 2022. Clin Transl Immunology. 11:e1413. PubMed
8. Masuta Y, *et al.* 2022. iScience. 25:105085. PubMed
9. Bekere I, *et al.* 2021. PLoS Pathog. 17:e1010074. PubMed
10. Wiernik A, *et al.* 2013. Clin Cancer Res. 19:3844. PubMed
11. Bratcher P, *et al.* 2016. J Cystic Fibrosis. 15: 67-73. PubMed
12. Hahn J, *et al.* 2019. Redox Biol. 26:101279. PubMed

RRID

AB_314217 (BioLegend Cat. No. 302017)
AB_314218 (BioLegend Cat. No. 302018)

Antigen Details

Structure Ig superfamily, transmembrane form (50-65 kD) or GPI-linked form (48 kD)

Distribution NK cells, activated monocytes, macrophages, neutrophils

Function Low affinity IgG Fc receptor, phagocytosis, ADCC

Ligand/Receptor Aggregated IgG, IgG-antigen complex

Cell Type Dendritic cells, Macrophages, Monocytes, Neutrophils, NK cells

Biology Area Immunology, Innate Immunity

Molecular Family CD Molecules, Fc Receptors

Antigen References

1. Fleit H, *et al.* 1982. *P. Natl. Acad. Sci. USA* 79:3275.
2. Stroncek D, *et al.* 1991. *Blood* 77:1572.
3. Wirthmueller U, *et al.* 1992. *J. Exp. Med.* 175:1381.

Gene ID 2214

Related Protocols

- Cell Surface Flow Cytometry Staining Protocol

Other Formats

APC anti-human CD16, Biotin anti-human CD16, FITC anti-human CD16, Brilliant Violet 711™ anti-human CD16, PE anti-human CD16, PE/Cyanine5 anti-human CD16, Purified anti-human CD16, APC/Cyanine7 anti-human CD16, PE/Cyanine7 anti-human CD16, Alexa Fluor® 488 anti-human CD16, Alexa Fluor® 647 anti-human CD16, Pacific Blue™ anti-human CD16, Alexa Fluor® 700 anti-human CD16, PerCP/Cyanine5.5 anti-human CD16, PerCP anti-human CD16, Brilliant Violet 421™ anti-human CD16,

Brilliant Violet 570™ anti-human CD16, Brilliant Violet 605™ anti-human CD16, Brilliant Violet 650™ anti-human CD16, Brilliant Violet 785™ anti-human CD16, Brilliant Violet 510™ anti-human CD16, Ultra-LEAF™ Purified anti-human CD16, Purified anti-human CD16 (Maxpar® Ready), PE/Dazzle™ 594 anti-human CD16, APC/Fire™ 750 anti-human CD16, TotalSeq™-A0083 anti-human CD16, TotalSeq™-B0083 anti-human CD16, TotalSeq™-C0083 anti-human CD16, PE/Fire™ 640 anti-human CD16, Spark YG™ 581 anti-human CD16, TotalSeq™-D0083 anti-human CD16, APC/Fire™ 810 anti-human CD16, GMP APC anti-human CD16, GMP PE/Dazzle™ 594 anti-human CD16, GMP PE anti-human CD16, Spark Red™ 718 anti-human CD16, GMP Pacific Blue™ anti-human CD16, GMP FITC anti-human CD16, Spark Blue™ 515 anti-human CD16, Spark UV™ 387 anti-human CD16, GMP PE/Cyanine7 anti-human CD16, GMP APC/Fire™ 750 anti-human CD16, Brilliant Violet 750™ anti-human CD16, Spark Blue™ 550 anti-human CD16, GMP PerCP/Cyanine5.5 anti-human CD16, Spark YG™ 593 anti-human CD16, Spark NIR™ 685 anti-human CD16, Spark Violet™ 500 anti-human CD16, Spark Blue™ 574 anti-human CD16 (Flexi-Fluor™), Spark PLUS UV395™ anti-human CD16, PerCP/Fire™ 806 anti-human CD16, PE/Fire™ 744 anti-human CD16 Antibody, PE/Fire™ 700 anti-human CD16 Antibody

Product Data

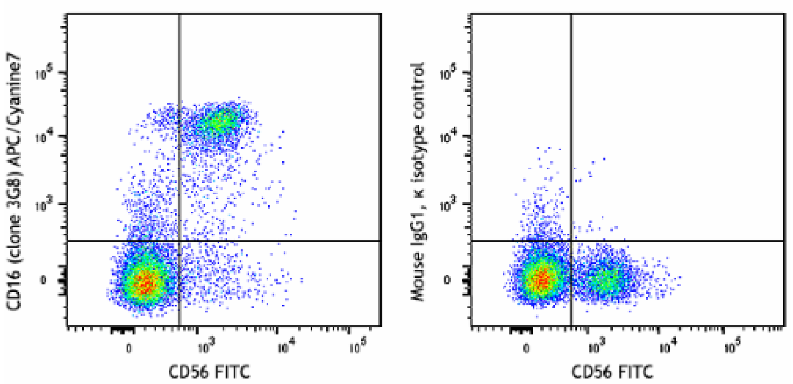

Human peripheral blood lymphocytes stained with CD56 (NCAM) FITC and CD16 (clone 3G8) APC/Cyanine7 (left), or Mouse IgG1, κ APC/Cyanine7 isotype (right).

For Research Use Only. Not for diagnostic or therapeutic use.

This product is supplied subject to the terms and conditions, including the limited license, located at www.biolegend.com/terms) ("Terms") and may be used only as provided in the Terms. Without limiting the foregoing, BioLegend products may not be used for any Commercial Purpose as defined in the Terms, resold in any form, used in manufacturing, or reverse engineered, sequenced, or otherwise studied or used to learn its design or composition without express written approval of BioLegend. Regardless of the information given in this document, user is solely responsible for determining any license requirements necessary for user's intended use and assumes all risk and liability arising from use of the product. BioLegend is not responsible for patent infringement or any other risks or liabilities whatsoever resulting from the use of its products.

BioLegend, the BioLegend logo, and all other trademarks are property of BioLegend, Inc. or their respective owners, and all rights are reserved.

8999 BioLegend Way, San Diego, CA 92121 www.biolegend.com
Toll-Free Phone: 1-877-Bio-Legend (246-5343) Phone: (858) 768-5800 Fax: (877) 455-9587

FITC anti-human CD86 Antibody

**Catalog# / Size**	374203 / 25 tests<br>374204 / 100 tests
**Clone**	BU63
**Regulatory Status**	RUO
**Workshop**	HCDM listed
**Other Names**	B7-2, B70, Ly-58
**Isotype**	Mouse IgG1, κ
**Description**	CD86 is an 80 kD immunoglobulin superfamily member also known as B7-2, B70, and Ly-58. CD86 is expressed on activated B and T cells, monocytes/macrophages, dendritic cells, and astrocytes. CD86, along with CD80, is the ligand of CD28 and CD152 (CTLA-4). CD86 is expressed earlier in the immune response than CD80. CD86 has also been shown to be involved in immunoglobulin class-switching and triggering of NK cell-mediated cytotoxicity. CD86 binds to CD28 to transduce costimulatory signals for T cell activation, proliferation, and cytokine production. CD86 can bind to CD152 as well, also known as CTLA-4, to deliver an inhibitory signal to T cells.

Product Details

**Verified Reactivity**	Human
**Antibody Type**	Monoclonal
**Host Species**	Mouse
**Immunogen**	ARH 77 (B lymphoblastoid cell line).
**Formulation**	Phosphate-buffered solution, pH 7.2, containing 0.09% sodium azide and BSA (origin USA)
**Preparation**	The antibody was purified by affinity chromatography and conjugated with FITC under optimal conditions.
**Concentration**	Lot-specific (to obtain lot-specific concentration and expiration, please enter the lot number in our Certificate of Analysis online tool.)
**Storage & Handling**	The antibody solution should be stored undiluted between 2°C and 8°C, and protected from prolonged exposure to light. **Do not freeze.**
**Application**	FC - *Quality tested*
**Recommended Usage**	Each lot of this antibody is quality control tested by immunofluorescent staining with flow cytometric analysis. For flow cytometric staining, the suggested use of this reagent is 5 µl per million cells in 100 µl staining volume or 5 µl per 100 µl of whole blood.
**Excitation Laser**	Blue Laser (488 nm)

Product Citations

1. Liu JJ, *et al.* 2022. Immun Inflamm Dis. 10:e735. PubMed
2. Miller CL, *et al.* 2022. Cell Chem Biol. 29:451. PubMed
3. Jiang Y, *et al.* 2023. Sci Adv. 9:eadc8933. PubMed
4. Toriyama M, *et al.* 2023. Front Mol Biosci. 10:1149828. PubMed
5. Yan F, *et al.* 2022. Cancer Discov. 12:792. PubMed
6. Vereertbrugghen A, *et al.* 2021. Front Oncol. 11:598319. PubMed
7. M?czy?ska J, *et al.* 2020. Cell Death Dis. 1.073611111. PubMed
8. Guo Z, *et al.* 2022. NPJ Precis Oncol. 6:34. PubMed
9. Rao X, *et al.* 2022. Cell Death Dis. 13:891. PubMed
10. Obradovic A, *et al.* 2021. Cell. 184(11):2988-3005.e16. PubMed
11. Jost M, *et al.* 2021. eLife. 10:00. PubMed
12. Bourdely P, *et al.* 2020. Immunity. 53(2):335-352. PubMed

RRID AB_2721573 (BioLegend Cat. No. 374203)
AB_2721574 (BioLegend Cat. No. 374204)

Antigen Details

Structure Ig superfamily, single-chain transmembrane glycoprotein, 80 kD

Distribution Monocytes/macrophages, activated B cells and T cells, dendritic cells

Function T Cell activation

Interaction C-Jun, NFκB

Ligand/Receptor CD28, CD152

Cell Type B cells, Dendritic cells, Macrophages, Monocytes, T cells

Biology Area Cell Biology, Costimulatory Molecules, Immunology, Neuroscience, Neuroscience Cell Markers

Molecular Family CD Molecules, Immune Checkpoint Receptors

Antigen References 1. Hathcock K, *et al.* 1996. *Adv. Immunol.* 62:131.
2. June C, *et al.* 1994. *Immunol. Today* 15:321.

Gene ID 942

Related Protocols

- Cell Surface Flow Cytometry Staining Protocol

Other Formats

Purified anti-human CD86, FITC anti-human CD86, PE anti-human CD86, APC anti-human CD86, PE/Cyanine7 anti-human CD86, Brilliant Violet 421™ anti-human CD86, Brilliant Violet 605™ anti-human CD86, PerCP/Cyanine5.5 anti-human CD86, PE/Dazzle™ 594 anti-human CD86, Spark YG™ 581 anti-human CD86 (Flexi-Fluor™), Spark YG™ 593 anti-human CD86 (Flexi-Fluor™) Antibody, Spark NIR™ 685 anti-human CD86 (Flexi-Fluor™) Antibody, Spark UV™ 387 anti-human CD86 (Flexi-Fluor™)

Product Data

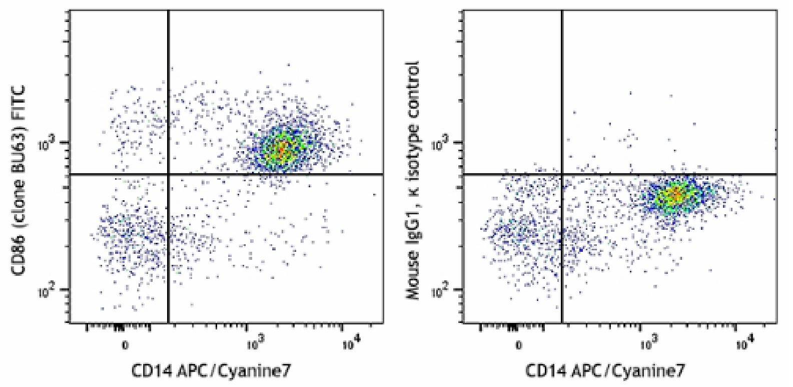

Human peripheral blood monocytes stained with CD14 APC/Cyanine7 and human CD86 (clone BU63, top) FITC or Mouse IgG1, κ FITC isotype control (bottom).

For Research Use Only. Not for diagnostic or therapeutic use.

This product is supplied subject to the terms and conditions, including the limited license, located at www.biolegend.com/terms) ("Terms") and may be used only as provided in the Terms. Without limiting the foregoing, BioLegend products may not be used for any Commercial Purpose as defined in the Terms, resold in any form, used in manufacturing, or reverse engineered, sequenced, or otherwise studied or used to learn its design or composition without express written approval of BioLegend. Regardless of the information given in this document, user is solely responsible for determining any license requirements necessary for user’s intended use and assumes all risk and liability arising from use of the product. BioLegend is not responsible for patent infringement or any other risks or liabilities whatsoever resulting from the use of its products.

BioLegend, the BioLegend logo, and all other trademarks are property of BioLegend, Inc. or their respective owners, and all rights are reserved.

8999 BioLegend Way, San Diego, CA 92121 www.biolegend.com
Toll-Free Phone: 1-877-Bio-Legend (246-5343) Phone: (858) 768-5800 Fax: (877) 455-9587

PE anti-human CD80 Recombinant Antibody

**Catalog# / Size**	370611 / 25 tests<br>370612 / 100 tests
**Clone**	QA18A16
**Regulatory Status**	RUO
**Other Names**	B7-1, B7, BB1
**Isotype**	Mouse IgG1, κ
**Description**	CD80, also known as B7-1, B7, and BB1, is a 60 kD single chain type I glycoprotein belonging to the immunoglobulin superfamily. CD80 is expressed on activated B and T cells, macrophages, and dendritic cells. CD80 binds to CD28 and CD152 (CTLA-4). Along with CD86, CD80 plays a critical role in regulation of T cell activation. The interaction of CD80 with CD28 provides a potent costimulatory signal for T cell activation through the CD3 complex, while its interaction with CTLA-4 provides an inhibitory signal for T cell activation.

Product Details

**Verified Reactivity**	Human
**Antibody Type**	Recombinant
**Host Species**	Mouse
**Formulation**	Phosphate-buffered solution, pH 7.2, containing 0.09% sodium azide and BSA (origin USA)
**Preparation**	The antibody was purified by affinity chromatography and conjugated with PE under optimal conditions.
**Concentration**	Lot-specific (to obtain lot-specific concentration and expiration, please enter the lot number in our Certificate of Analysis online tool.)
**Storage & Handling**	The antibody solution should be stored undiluted between 2°C and 8°C, and protected from prolonged exposure to light. **Do not freeze**.
**Application**	FC - Quality tested
**Recommended Usage**	Each lot of this antibody is quality control tested by immunofluorescent staining with flow cytometric analysis. For flow cytometric staining, the suggested use of this reagent is 5 µL per million cells in 100 µL staining volume or 5 µL per 100 µL of whole blood. It is recommended that the reagent be titrated for optimal performance for each application.
**Excitation Laser**	Blue Laser (488 nm)<br>Green Laser (532 nm)/Yellow-Green Laser (561 nm)
**RRID**	AB_2890803 (BioLegend Cat. No. 370611)<br>AB_2890803 (BioLegend Cat. No. 370612)

Antigen Details

**Structure**	Ig superfamily, single chain type I transmembrane glycoprotein, 60 kD
**Function**	T cell costimulation
**Ligand/Receptor**	CD28, CD152 (CTLA-4)
**Cell Type**	B cells, Dendritic cells, Macrophages, T cells, Tregs
**Biology Area**	Cell Biology, Costimulatory Molecules, Immunology, Neuroscience, Neuroscience Cell Markers

**Molecular Family**	CD Molecules, Immune Checkpoint Receptors
**Antigen References**	1. Kim GJ, *et al*. 2012. *Immunol*. 188:4217-4225.
**Gene ID**	941

Related Protocols

- Cell Surface Flow Cytometry Staining Protocol

Other Formats

PE anti-human CD80 Recombinant Antibody, Purified anti-human CD80 Recombinant Antibody, Ultra-LEAF™ Purified anti-human CD80 Recombinant Antibody

Product Data

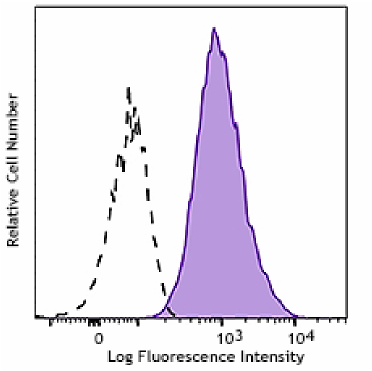

Human B-cell Burkitt's lymphoma cell line Raji was stained with CD80 recombinant (clone QA18A16) PE (filled histogram) or mouse IgG1, κ PE isotype control (open histogram).

For Research Use Only. Not for diagnostic or therapeutic use.

This product is supplied subject to the terms and conditions, including the limited license, located at www.biolegend.com/terms) ("Terms") and may be used only as provided in the Terms. Without limiting the foregoing, BioLegend products may not be used for any Commercial Purpose as defined in the Terms, resold in any form, used in manufacturing, or reverse engineered, sequenced, or otherwise studied or used to learn its design or composition without express written approval of BioLegend. Regardless of the information given in this document, user is solely responsible for determining any license requirements necessary for user's intended use and assumes all risk and liability arising from use of the product. BioLegend is not responsible for patent infringement or any other risks or liabilities whatsoever resulting from the use of its products.

BioLegend, the BioLegend logo, and all other trademarks are property of BioLegend, Inc. or their respective owners, and all rights are reserved.

8999 BioLegend Way, San Diego, CA 92121 www.biolegend.com
Toll-Free Phone: 1-877-Bio-Legend (246-5343) Phone: (858) 768-5800 Fax: (877) 455-9587

PE/Cyanine7 anti-human CD64 Antibody

**Catalog# / Size**	305021 / 25 tests<br>305022 / 100 tests
**Clone**	10.1
**Regulatory Status**	RUO
**Workshop**	VI MA36
**Other Names**	FcγRI, FcR I, FCGR1A
**Isotype**	Mouse IgG1, κ
**Description**	CD64 is a 72 kD single chain type I glycoprotein also known as FcγRI and FcR I. CD64 is a member of the immunoglobulin superfamily and is expressed on monocytes/macrophages, dendritic cells, and activated granulocytes. The expression can be upregulated by IFN-γ stimulation. CD64 binds IgG immune complex. It plays a role in antigen capture, phagocytosis of IgG/antigen complexes, and antibody-dependent cellular cytotoxicity (ADCC).

Product Details

**Verified Reactivity**	Human, Cynomolgus, Rhesus
**Reported Reactivity**	Baboon, Capuchin Monkey, Chimpanzee, Squirrel Monkey
**Antibody Type**	Monoclonal
**Host Species**	Mouse
**Immunogen**	Human rheumatoid synovial fluid cells and fibronectin-purified monocytes.
**Formulation**	Phosphate-buffered solution, pH 7.2, containing 0.09% sodium azide and BSA (origin USA)
**Preparation**	The antibody was purified by affinity chromatography and conjugated with PE/Cyanine7 under optimal conditions.
**Concentration**	Lot-specific (to obtain lot-specific concentration and expiration, please enter the lot number in our Certificate of Analysis online tool.)
**Storage & Handling**	The antibody solution should be stored undiluted between 2°C and 8°C, and protected from prolonged exposure to light. **Do not freeze.**
**Application**	FC - Quality tested
**Recommended Usage**	Each lot of this antibody is quality control tested by immunofluorescent staining with flow cytometric analysis. For flow cytometric staining, the suggested use of this reagent is 5 µl per million cells in 100 µl staining volume or 5 µl per 100 µl of whole blood.
**Excitation Laser**	Blue Laser (488 nm)<br>Green Laser (532 nm)/Yellow-Green Laser (561 nm)
**Application Notes**	Clone 10.1 recognizes the EC3 epitope of CD64. While both contain the EC3 domain, in-house testing suggests that clone 10.1 preferentially binds to CD64A (FcγRIA), but not CD64B (FcγRIB). Additional reported applications (for the relevant formats) include: blocking of human IgG3 and murine IgG2a binding to FcγRI[2,5,6,11] and immunohistochemical staining of acetone-fixed frozen tissue sections[12].

Application References

1. McMichael A, *et al.* Eds. 1987. Leucocyte Typing III. Oxford University Press. New York.
2. Schlossman S, *et al.* Eds. 1995. Leucocyte Typing V. Oxford University Press. New York. p. 874.
3. Kishimoto T, *et al.* Eds. 1997. Leucocyte Typing VI. Garland Publishing Inc. London.
4. Holl V, *et al.* 2004. *J. Immunol.* 173:6274.
5. Hober D, *et al.* 2002. *J. Gen. Virol.* 83:2169.
6. Cho HJ, *et al.* 2007. *Physiol Genomics* 149:60.
7. van Tits L, *et al.* 2005. *Arterioscler Thromb Vasc Biol.* 25:717. PubMed
8. Bruhns P, *et al.* 2008. *Blood* 113:3716. PubMed
9. Yoshino N, *et al.* 2000. *Exp. Anim. (Tokyo)* 49:97. (FC)
10. Carter DL, *et al.* 1999. *Cytometry* 37:41. (FC)
11. Dougherty GJ, *et al.* 1987. *Eur. J. Immunol.* 17:1453.
12. Blom AB, *et al.* 2003. *Arthritis Rheum.* 48(4):1002-14. (IHC)

Product Citations

1. Lee PY, *et al.* 2020. Journal of Allergy and Clinical Immunology. 146(5):1194-1200.e1. PubMed
2. Fatehi Hassanabad A, *et al.* 2022. JTCVS Open. 12:118. PubMed
3. Bsat M, *et al.* 2021. Curr Protoc. 1:e74. PubMed
4. Mann E, *et al.* 2015. Gut. . PubMed
5. Nowak W, *et al.* 2020. EBioMedicine. 50:290-305.. PubMed
6. Keck S, *et al.* 2021. Cellular and Molecular Gastroenterology and Hepatology. 12(2):507-545. PubMed
7. Bailey AL, *et al.* 2020. bioRxiv. . PubMed

RRID AB_2561583 (BioLegend Cat. No. 305021)
AB_2561584 (BioLegend Cat. No. 305022)

Antigen Details

Structure Ig superfamily, type I glycoprotein, 72 kD

Distribution Monocytes, macrophages, dendritic cells, activated granulocytes

Function Phagocytosis, ADCC

Ligand/Receptor IgG receptor

Cell Type Dendritic cells, Granulocytes, Macrophages, Monocytes

Biology Area Immunology, Innate Immunity

Molecular Family CD Molecules, Fc Receptors

Antigen References

1. Hulett M, *et al.* 1994. *Adv. Immunol.* 57:1.
2. van de Winkel J, *et al.* 1993. *Immunol. Today* 14:215.

Gene ID 2209

Related Protocols

- Cell Surface Flow Cytometry Staining Protocol

Other Formats

Biotin anti-human CD64, FITC anti-human CD64, PE anti-human CD64, Purified anti-human CD64, Alexa Fluor® 488 anti-human CD64, Alexa Fluor® 647 anti-human CD64, APC anti-human CD64, Pacific Blue™ anti-human CD64, Brilliant Violet 421™ anti-human CD64, PE/Cyanine7 anti-human CD64, PerCP/Cyanine5.5 anti-human CD64, APC/Cyanine7 anti-human CD64, Brilliant Violet 510™ anti-human CD64, Purified anti-human CD64 (Maxpar® Ready), PE/Dazzle™ 594 anti-human CD64, Brilliant Violet 605™ anti-human CD64, APC/Fire™ 750 anti-human CD64, TotalSeq™-A0162 anti-human CD64, Brilliant Violet 711™ anti-human CD64, Alexa Fluor® 700 anti-human CD64, Brilliant Violet 785™ anti-human CD64, TotalSeq™-C0162 anti-human CD64, Ultra-LEAF™ Purified anti-human CD64, TotalSeq™-B0162 anti-human CD64, TotalSeq™-D0162 anti-human CD64, GMP PE anti-human CD64, GMP FITC anti-human CD64, Brilliant Violet 650™ anti-human CD64, GMP PE/Dazzle™ 594 anti-human CD64, GMP APC/Fire™ 750 anti-human CD64, GMP PE/Cyanine7 anti-human CD64, GMP PerCP/Cyanine5.5 anti-human CD64

Product Data

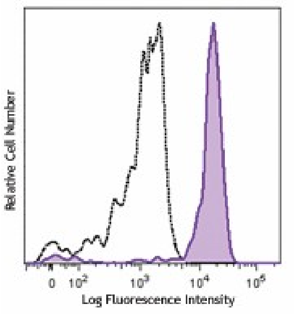

Human peripheral blood monocytes stained with anti-human CD64 (clone 10.1) PE/Cyanine7 (filled histogram) or mouse IgG1, κ PE/Cyanine7 isotype control (open histogram).

For Research Use Only. Not for diagnostic or therapeutic use.

This product is supplied subject to the terms and conditions, including the limited license, located at www.biolegend.com/terms) ("Terms") and may be used only as provided in the Terms. Without limiting the foregoing, BioLegend products may not be used for any Commercial Purpose as defined in the Terms, resold in any form, used in manufacturing, or reverse engineered, sequenced, or otherwise studied or used to learn its design or composition without express written approval of BioLegend. Regardless of the information given in this document, user is solely responsible for determining any license requirements necessary for user's intended use and assumes all risk and liability arising from the use of the product. BioLegend is not responsible for patent infringement or any other risks or liabilities whatsoever resulting from the use of its products.

BioLegend, the BioLegend logo, and all other trademarks are property of BioLegend, Inc. or their respective owners, and all rights are reserved.

8999 BioLegend Way, San Diego, CA 92121 www.biolegend.com
Toll-Free Phone: 1-877-Bio-Legend (246-5343) Phone: (858) 768-5800 Fax: (877) 455-9587

BioLegend®

PerCP/Cyanine5.5 anti-human CD14 Antibody

**Catalog# / Size**	325621 / 25 tests<br>325622 / 100 tests
**Clone**	HCD14
**Regulatory Status**	RUO
**Other Names**	LPS receptor
**Isotype**	Mouse IgG1, κ
**Description**	CD14 is a 53-55 kD glycosylphosphatidylinositol (GPI)-linked membrane glycoprotein also known as LPS receptor. CD14 is expressed at high levels on monocytes and macrophages, and at lower levels on granulocytes. Some dendritic cell populations such as interfollicular dendritic cells, reticular dendritic cells, and Langerhans cells have also been reported to express CD14. As a high-affinity receptor for LPS, CD14 is involved in the clearance of gram-negative pathogens and in the upregulation of adhesion molecules and cytokines expression in monocytes and neutrophils.

Product Details

**Verified Reactivity**	Human
**Antibody Type**	Monoclonal
**Host Species**	Mouse
**Formulation**	Phosphate-buffered solution, pH 7.2, containing 0.09% sodium azide and BSA (origin USA)
**Preparation**	The antibody was purified by affinity chromatography, and conjugated with PerCP/Cyanine5.5 under optimal conditions.
**Concentration**	Lot-specific (to obtain lot-specific concentration and expiration, please enter the lot number in our Certificate of Analysis online tool.)
**Storage & Handling**	The CD14 antibody solution should be stored undiluted between 2°C and 8°C, and protected from prolonged exposure to light. **Do not freeze.**
**Application**	FC - Quality tested
**Recommended Usage**	Each lot of this antibody is quality control tested by immunofluorescent staining with flow cytometric analysis. For flow cytometric staining, the suggested use of this reagent is 5 µl per million cells in 100 µl staining volume or 5 µl per 100 µl of whole blood.<br><br>* PerCP/Cyanine5.5 has a maximum absorption of 482 nm and a maximum emission of 690 nm.
**Excitation Laser**	Blue Laser (488 nm)
**Application Notes**	Additional reported applications (for the relevant formats) include: immunofluorescence microscopy. This clone was tested in-house and does not work on formalin fixed paraffin-embedded (FFPE) tissue.

Application References

1. McMichael A, *et al.* 1987. Leucocyte Typing III. Oxford University Press. New York.
2. Knapp W, *et al.* Eds. 1989. Leucocyte Typing IV. Oxford University Press. New York.
3. Schlossman S, *et al.* Eds. 1995. Leucocyte Typing V. Oxford University Press. New York.

Product Citations

1. Kenney DJ, *et al.* 2022. Cell Rep. 39:110714. PubMed
2. Lertjuthaporn S, *et al.* 2022. Pathogens. 11: . PubMed
3. Höfle J, *et al.* 2022. EMBO Rep. 23:e54133. PubMed
4. Barnes CO, *et al.* 2022. Sci Adv. 8:eabp8155. PubMed
5. Porbahaie M, *et al.* 2023. PLoS One. 18:e0279626. PubMed
6. Sposito B, *et al.* 2021. Cell. 184:4953. PubMed
7. Zhou R, *et al.* 2020. Immunity. S1074-7613(20)30333-2.. PubMed
8. Kimura I, *et al.* 2022. Cell Rep. 38:110218. PubMed
9. Weisberg SP, *et al.* 2020. Cell Reports. 29(12):3916-3932.e5.. PubMed
10. Jacobs ME, *et al.* 2021. Immunother Adv. 1:ltaa004. PubMed
11. Ma L, *et al.* 2021. Clinical Cancer Research. 27(6):1778-1791. PubMed
12. Antonucci L, *et al.* 2020. Journal of Immunology Research. 3257.929861. PubMed

RRID AB_893252 (BioLegend Cat. No. 325621)
AB_893250 (BioLegend Cat. No. 325622)

Antigen Details

Structure GPI-linked membrane glycoprotein, 53-55 kD

Distribution Monocytes, macrophages, granulocytes (low)

Function LPS receptor, clearance of Gram-negative pathogens

Ligand/Receptor LPS

Cell Type Granulocytes, Macrophages, Monocytes, Neutrophils

Biology Area Cell Biology, Immunology, Innate Immunity, Neuroinflammation, Neuroscience

Molecular Family CD Molecules

Antigen References 1. Stocks S, *et al.* 1990. *Biochem. J.* 268:275.
2. Wright S, *et al.* 1990. *Science* 249:1434.

Gene ID 929

Related Protocols

- Cell Surface Flow Cytometry Staining Protocol

Other Formats

PerCP anti-human CD14, Purified anti-human CD14, FITC anti-human CD14, PE anti-human CD14, APC anti-human CD14, Alexa Fluor® 488 anti-human CD14, Alexa Fluor® 647 anti-human CD14, Alexa Fluor® 700 anti-human CD14, Pacific Blue™ anti-human CD14, PE/Cyanine7 anti-human CD14, APC/Cyanine7 anti-human CD14, PerCP/Cyanine5.5 anti-human CD14, Biotin anti-human CD14, Brilliant Violet 421™ anti-human CD14, Alexa Fluor® 594 anti-human CD14, PE/Dazzle™ 594 anti-human CD14, Spark Blue™ 574 anti-human CD14, Brilliant Violet 510™ anti-human CD14, Brilliant Violet 605™ anti-human CD14, Brilliant Violet 650™ anti-human CD14, Brilliant Violet 785™ anti-human CD14, StarBright UltraViolet 740 anti-human CD14

Product Data

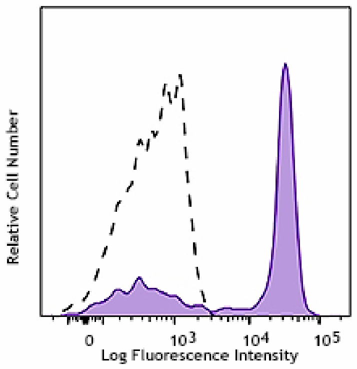

Human peripheral blood monocytes were stained with CD14 (Clone HCD14) PerCP/Cyanine5.5 (filled histogram), or mouse IgG1, κ PerCP/Cyanine5.5 isotype control (open histogram).

For Research Use Only. Not for diagnostic or therapeutic use.

This product is supplied subject to the terms and conditions, including the limited license, located at www.biolegend.com/terms) ("Terms") and may be used only as provided in the Terms. Without limiting the foregoing, BioLegend products may not be used for any Commercial Purpose as defined in the Terms, resold in any form, used in manufacturing, or reverse engineered, sequenced, or otherwise studied or used to learn its design or composition without express written approval of BioLegend. Regardless of the information given in this document, user is solely responsible for determining any license requirements necessary for user's intended use and assumes all risk and liability arising from use of the product. BioLegend is not responsible for patent infringement or any other risks or liabilities whatsoever resulting from the use of its products.

BioLegend, the BioLegend logo, and all other trademarks are property of BioLegend, Inc. or their respective owners, and all rights are reserved.

8999 BioLegend Way, San Diego, CA 92121 www.biolegend.com
Toll-Free Phone: 1-877-Bio-Legend (246-5343) Phone: (858) 768-5800 Fax: (877) 455-9587
