# Supplementary material for: Treatment in acute HIV infection only temporarily preserves monocyte function: a comparative cohort study in adult males
Source: eBioMedicine. 2025 Nov 7;122:105997. doi: 10.1016/j.ebiom.2025.105997 (PMC12790590; doi:10.1016/j.ebiom.2025.105997)
Supplement: NOVA board team [file mmc3.docx]

NOVA board team

| **First names** | **Surnames** |
| --- | --- |
| Godelieve | De Bree |
| Jan M | Prins |
| Annelies | Verbon |
| Liffert | Vogt |
| Peter | Reiss |
| Casper | Rokx |
