# Supplementary material for: Treatment in acute HIV infection only temporarily preserves monocyte function: a comparative cohort study in adult males
Source: eBioMedicine. 2025 Nov 7;122:105997. doi: 10.1016/j.ebiom.2025.105997 (PMC12790590; doi:10.1016/j.ebiom.2025.105997)
Supplement: Supplemental Figures Plus Legends [file mmc4.pdf]

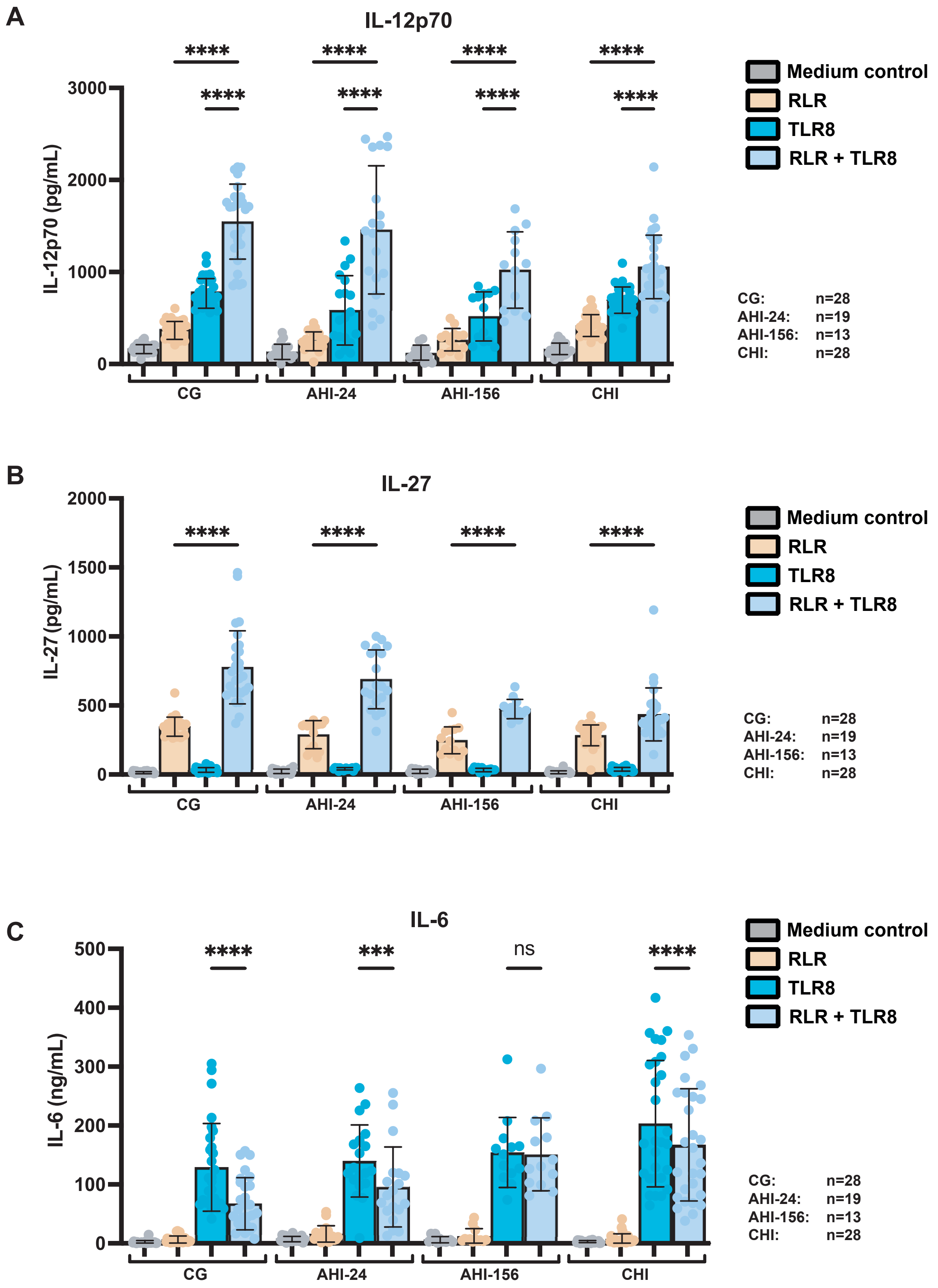

Figure s1: TLR8 and RLR agonists induce IL-12p70, IL-27 and IL-6 with crosstalk inducing IL-12p70 and IL-27 while restricting IL-6. Prolonged HIV-1 infection impairs this crosstalk.

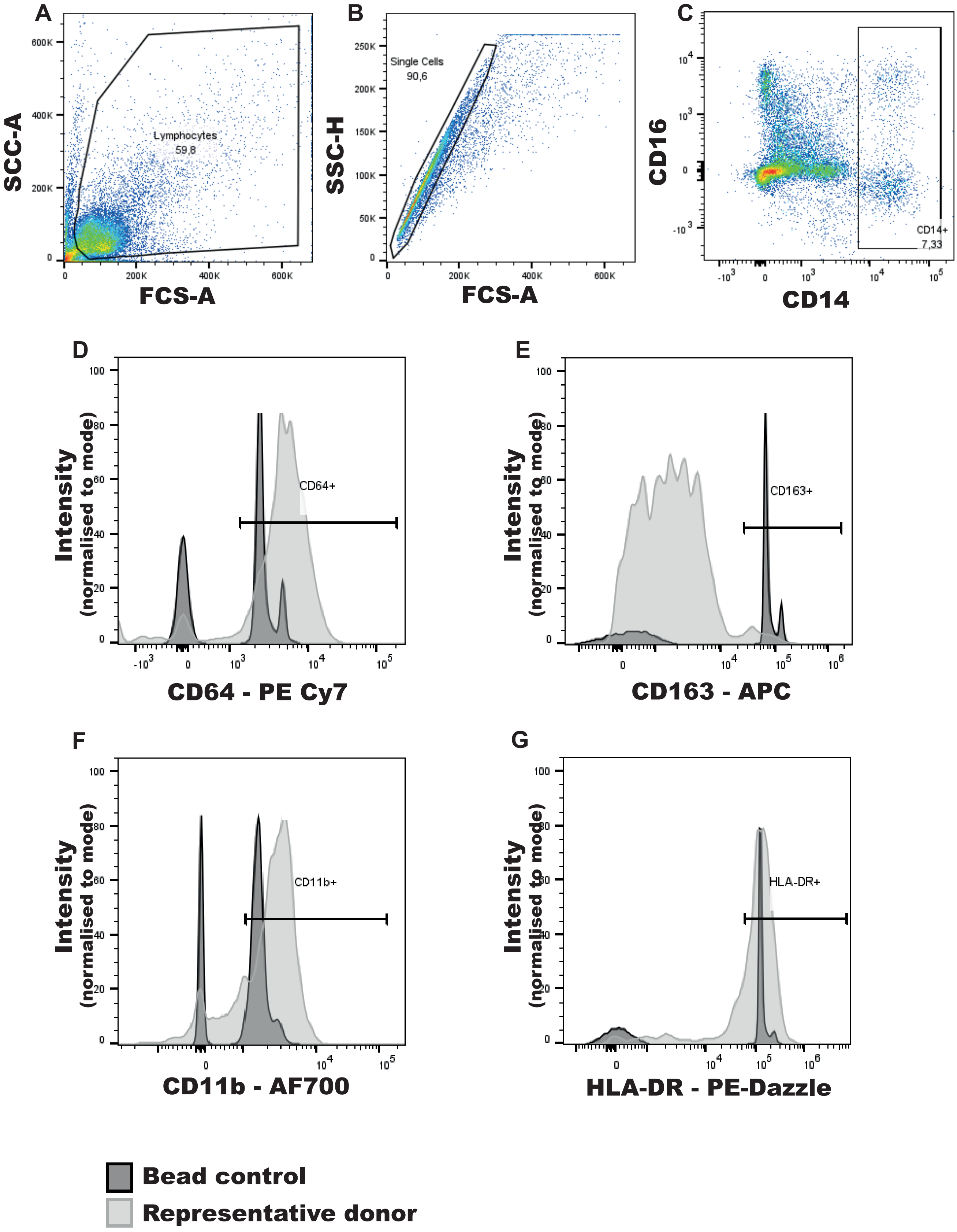

Figure s2: Gating strategy

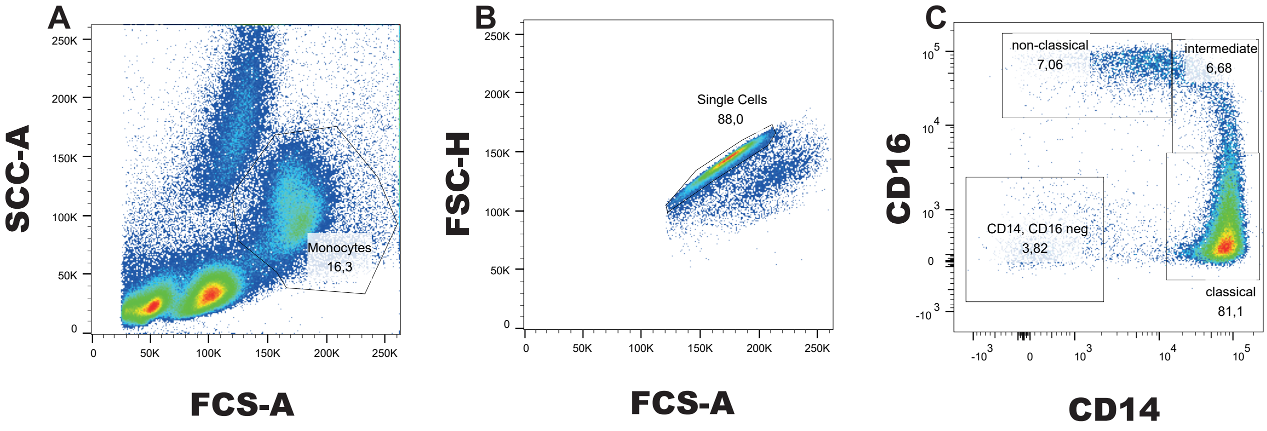

Figure s3: Gating strategy matched AHI participants.

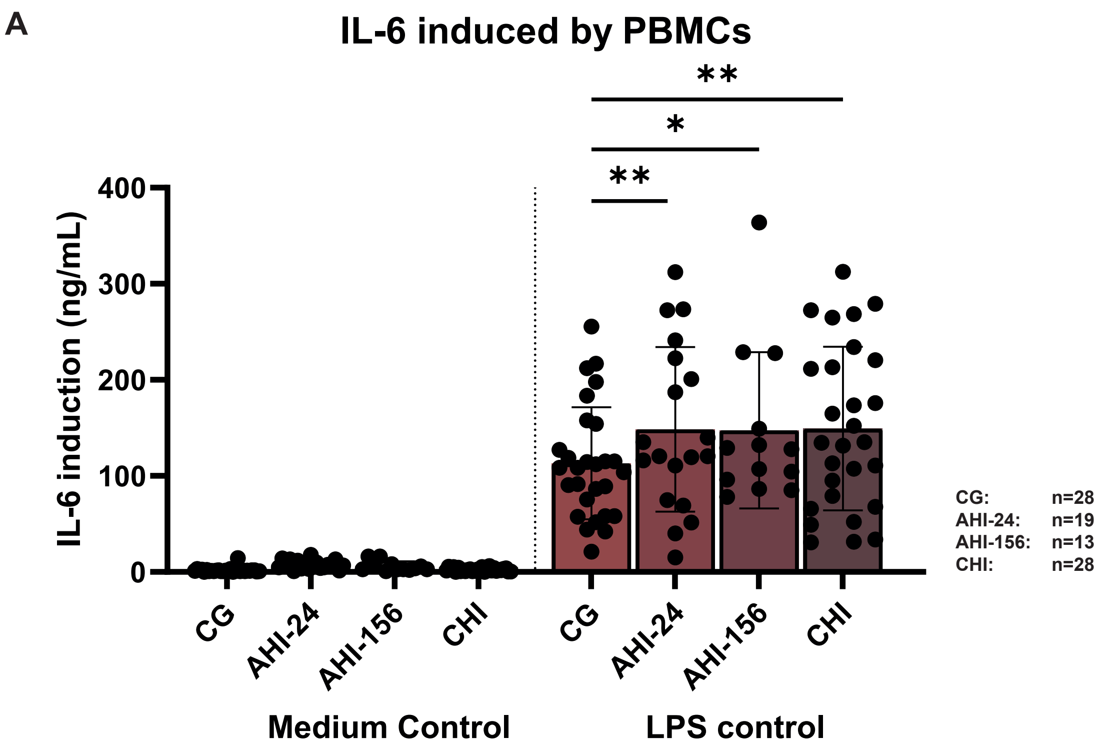

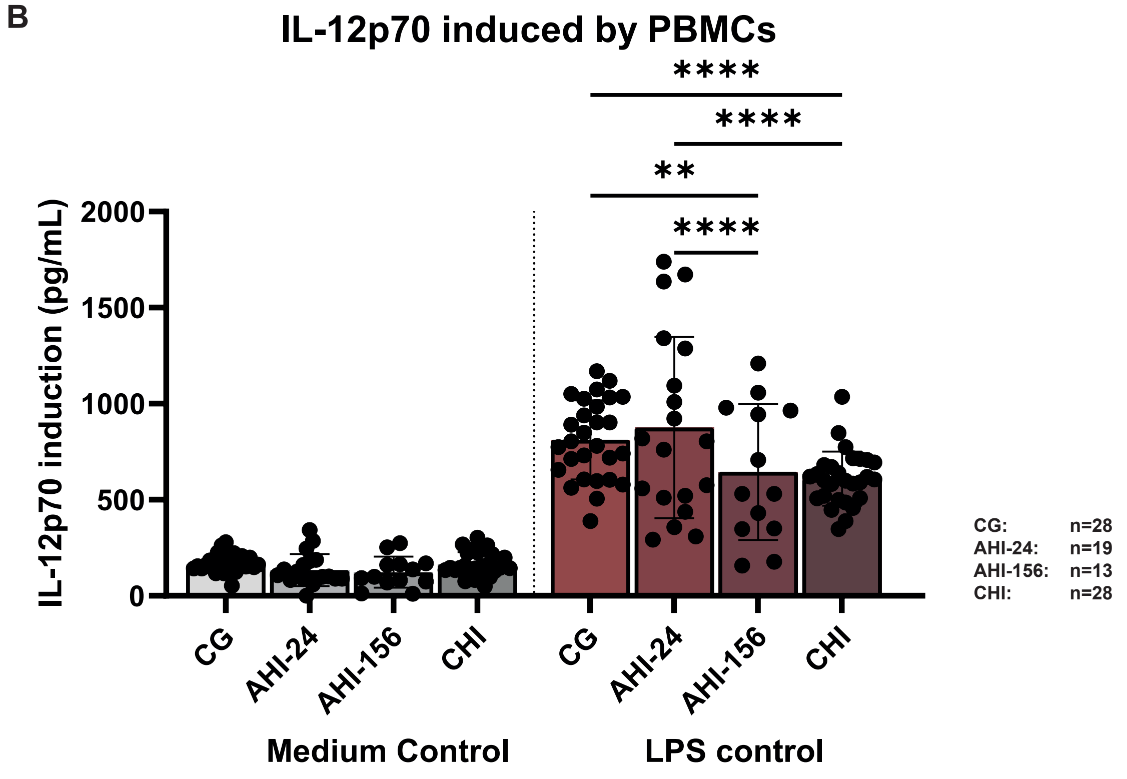

Figure s4: IL-6 and IL-12p70 induction to LPS internal controls

Supplemental Figure s1: TLR8 and RLR co-stimulation induces distinct cytokine profiles in HIV-negative individuals and dysregulated responses in CHI and AHI groups.
(A-C) PBMCs from CG, AHI-24, AHI-156, and CHI individuals were stimulated with TLR8 and RLR agonists, and cytokine responses were assessed by ELISA. CG n=28, AHI-24 n=19, AHI-156 n=13, CHI n=28. Three biological replicates were used per datapoint. Error bars show SD.
Statistical analysis was performed using paired two-way ANOVA with Tukey's post-hoc correction. Significance is indicated as *$P < 0.05$, **$P < 0.01$, ***$P < 0.001$, ****$P < 0.0001$.

Supplemental Figure s2: Gating strategy
Surface expression of CD11b, CD64, CD163, and HLA-DR was assessed on monocytes from CG, CHI, AHI-24, and AHI-156 groups.
Cells were thawed. A lymphocyte gate was applied based on forward/sideward scatter (A), subsequently a single-cell gate was set (B). Classical and intermediate monocytes were isolated for CD14 expression (C). Positive samples were determined by single staining on control beads (D-G).

Supplemental Figure s3: Gating strategy matched AHI participants.
A monocyte gate was applied based on forward/sideward scatter (A), subsequently a single-cell gate was set (B). Monocyte subsets were determined based on CD14 and CD16 expression (C): Classical monocytes (CD14++/CD16-), intermediate monocytes (CD14++,CD16+) and non-classical monocytes (CD14+,CD16++). Positive samples were determined by single staining on control beads.

Supplemental Figure s4: Upregulation of pro-inflammatory cytokines in monocytes from CHI with FDR testing.
PBMCs from CG, AHI-24, AHI-156, and CHI individuals were stimulated with LPS at 10ng/mL, and (A) IL-6 and (B) IL-12p70 secretion in the supernatant after 24h was assessed by ELISA. CG n=28, AHI-24 n=19, AHI-156 n=13, CHI n=28. Three biological replicates were used per datapoint. Error bars show SD.
Statistical analysis was performed using unpaired two-way ANOVA with Tukey's post-hoc correction. Significance is indicated as *$P < 0.05$, **$P < 0.01$, ***$P < 0.001$, ****$P < 0.0001$.
